# The Role of Regulator Catabolite Control Protein A (CcpA) in Streptococcus agalactiae Physiology and Stress Response

**DOI:** 10.1128/spectrum.02080-22

**Published:** 2022-10-20

**Authors:** Anne-Emmanuelle Roux, Sylvie Robert, Mathilde Bastat, Isabelle Rosinski-Chupin, Vanessa Rong, Sébastien Holbert, Laurent Mereghetti, Emilie Camiade

**Affiliations:** a ISP, Université de Tours, INRAE, Tours, France; b Unité Écologie et Évolution de la Résistance aux Antibiotiques, CNRS UMR3525, Institut Pasteur, Paris, France; c CHRU Tours, Service de Bactériologie-Virologie-Hygiène, Tours, France; Griffith University

**Keywords:** carbon catabolite repression (CCR), CcpA, stress, adaptation, *Streptococcus agalactiae*, Usp proteins, acid resistance, catabolite repression, group B *Streptococcus*, oxidative stress, stress adaptation, stress response

## Abstract

Streptococcus agalactiae is a leading cause of infections in neonates. This opportunistic pathogen colonizes the vagina, where it has to cope with acidic pH and hydrogen peroxide produced by lactobacilli. Thus, in the host, this bacterium possesses numerous adaptation mechanisms in which the pleiotropic regulators play a major role. The transcriptional regulator CcpA (catabolite control protein A) has previously been shown to be the major regulator involved in carbon catabolite repression in Gram-positive bacteria but is also involved in other functions. By transcriptomic analysis, we characterized the CcpA-dependent gene regulation in S. agalactiae. Approximately 13.5% of the genome of S. agalactiae depends on CcpA for regulation and comprises genes involved in sugar uptake and fermentation, confirming the role of CcpA in carbon metabolism. We confirmed by electrophoretic mobility shift assays (EMSAs) that the DNA binding site called *cis*-acting catabolite responsive element (*cre*) determined for other streptococci was effective in S. agalactiae. We also showed that CcpA is of capital importance for survival under acidic and oxidative stresses and is implicated in macrophage survival by regulating several genes putatively or already described as involved in stress response. Among them, we focused our study on *SAK_1689*, which codes a putative UspA protein. We demonstrated that *SAK_1689*, highly downregulated by CcpA, is overexpressed under oxidative stress conditions, this overexpression being harmful for the bacterium in a Δ*ccpA* mutant.

**IMPORTANCE** Streptococcus agalactiae is a major cause of disease burden leading to morbidity and mortality in neonates worldwide. Deciphering its adaptation mechanisms is essential to understand how this bacterium manages to colonize its host. Here, we determined the regulon of the pleiotropic regulator CcpA in S. agalactiae. Our findings reveal that CcpA is not only involved in carbon catabolite repression, but is also important for acidic and oxidative stress resistance and survival in macrophages.

## INTRODUCTION

The commensal acterium Streptococcus agalactiae, also referred as group B Streptococcus, is a Gram-positive bacterium with a broad spectrum of hosts. First discovered as an agent of bovine mastitis (1), it is now known to be a cause of infections in different animals and especially in the farm fish leading to economic losses (2–4). This bacterium is also a leading cause of neonatal infections and an emerging pathogen in elderly and immunocompromised adults (5–7). S. agalactiae, a commensal of the gastrointestinal and genitourinary tracts, is found in pregnant women worldwide (11 to 35%) (8). During delivery, it can be transmitted from the mother to the child by aspiration of vaginal and/or amniotic fluids, leading to pneumonia and eventually bacteremia and meningitis if it manages to reach the blood (5). This bacterium is responsible each year for approximately 91,000 neonatal deaths and 37,000 survivors with neurodevelopmental impairment worldwide. Moreover, *in utero* infections are responsible for 46,000 stillbirths and could be responsible for some 518,000 preterm births each year (9).

In the host, S. agalactiae has to adapt to variations in physicochemical conditions. As an example, when S. agalactiae colonizes the vagina, it is confronted with oxidative stress and acidic pH (pH < 4.5). Indeed, lactobacilli, which are part of the vaginal flora, produce hydrogen peroxide and organic acids that lower the vaginal pH (10). Bacteria also undergo oxidative stress because of their own metabolism. Stress resistance mechanisms are also useful in resisting the innate immune system, particularly phagocytosis by macrophages and neutrophils. After being phagocytosed, pathogens are incorporated into a phagosome which matures in a highly microbicidal organelle thanks to acidic pH, production of reactive oxygen species (ROS), reactive nitrogen species (RNS), and antimicrobial peptides (11). It has been shown that S. agalactiae is able to survive in the mature phagolysosome for an extended period of time (12–15). Thus, to cope with low pH, S. agalactiae possesses numerous defense mechanisms (16, 17) that are regulated by two-component systems and transcriptional regulators (16, 18, 19). The oxidative stress response also contributes to acid adaptation (16) and occurs when ROS production prevails over the bacterium’s ability to remove the threat (20). Oxidative stress can result in damage to nucleic acids, amino acids, cofactors of proteins, and lipids, and it is counteracted thanks to various mechanisms (17, 20–27).

The variety of hosts and sites that this bacterium can colonize demonstrates its ability to adapt and, especially, its capacity to acquire nutrients such as sugars from its environment. S. agalactiae possesses a high number of sugar transporters; in particular, phosphotransferase systems (PTS) that play an important role in carbon catabolite repression (CCR) attest to this adaptability (28). The inflow of sugars into the cell and their catabolism are regulated by CCR. This mechanism avoids the waste of energy by prioritizing the use of sugars that are rapidly metabolizable. In Bacillus subtilis, CCR occurs when the presence of a rapidly metabolizable sugar in the medium leads to the formation of a complex of two proteins, the coeffector phosphoprotein HPr-Ser-46-P and CcpA, a pleiotropic transcriptional regulator of the LacI/GalR family (29, 30). In *Firmicutes*, CcpA binds to a *cis*-acting catabolite responsive element (*cre*) DNA sequence enabling the regulation of approximatively 10 to 20% of the genome (31–34). In B. subtilis, these sequences are imperfect palindromes with the consensus sequence WTGNNARCGNWWWCAW (35–38). CcpA is thought to act as a repressor when *cre* sites are located upstream of the promoter region and as an activator when *cre* sites are located downstream. However, in B. subtilis, *in silico* analyses have shown the presence of *cre* sites in most operons subject to CCR via CcpA, but a few *cre* sites have been found in the genes submitted to catabolic activation via CcpA (31, 39). The CcpA regulon has been studied in a few species of streptococci, highlighting its major role in carbohydrate metabolism (33, 40–42). In group A Streptococcus it regulates between 20 and 30% of the genome depending on the study (40, 41). In Streptococcus suis it regulates approximately 10% of the genome (33), whereas in Streptococcus mutans it regulates only 48 genes out of 1,960 (42). Other than carbon metabolism, CcpA has also been implicated in the regulation of other functions, such as virulence or stress response (33, 40, 41, 43). In S. agalactiae, the role of CcpA has not yet been described but a study has described *ccpA* as an essential gene (44).

In this study, we determined the regulon of CcpA in S. agalactiae by transcriptomic analysis. We then performed *in silico* analysis to identify putative *cre* sites in the S. agalactiae genome and experimentally confirmed the *cre* consensus sequence predicted *in silico* by electrophoretic mobility shift assays (EMSAs). Among the CcpA regulated genes, up to 12 genes were potentially involved in the stress response. We showed that CcpA contributed to S. agalactiae survival under acidic and oxidative conditions and for surviving inside macrophages. The role of two putative universal stress proteins (UspA), SAK_1689, whose encoding gene is strongly regulated by CcpA in the transcriptome, and SAK_1741, was assessed under stress conditions.

## RESULTS

### An eighth of the S. agalactiae genome is regulated by CcpA.

**FIG 1:**
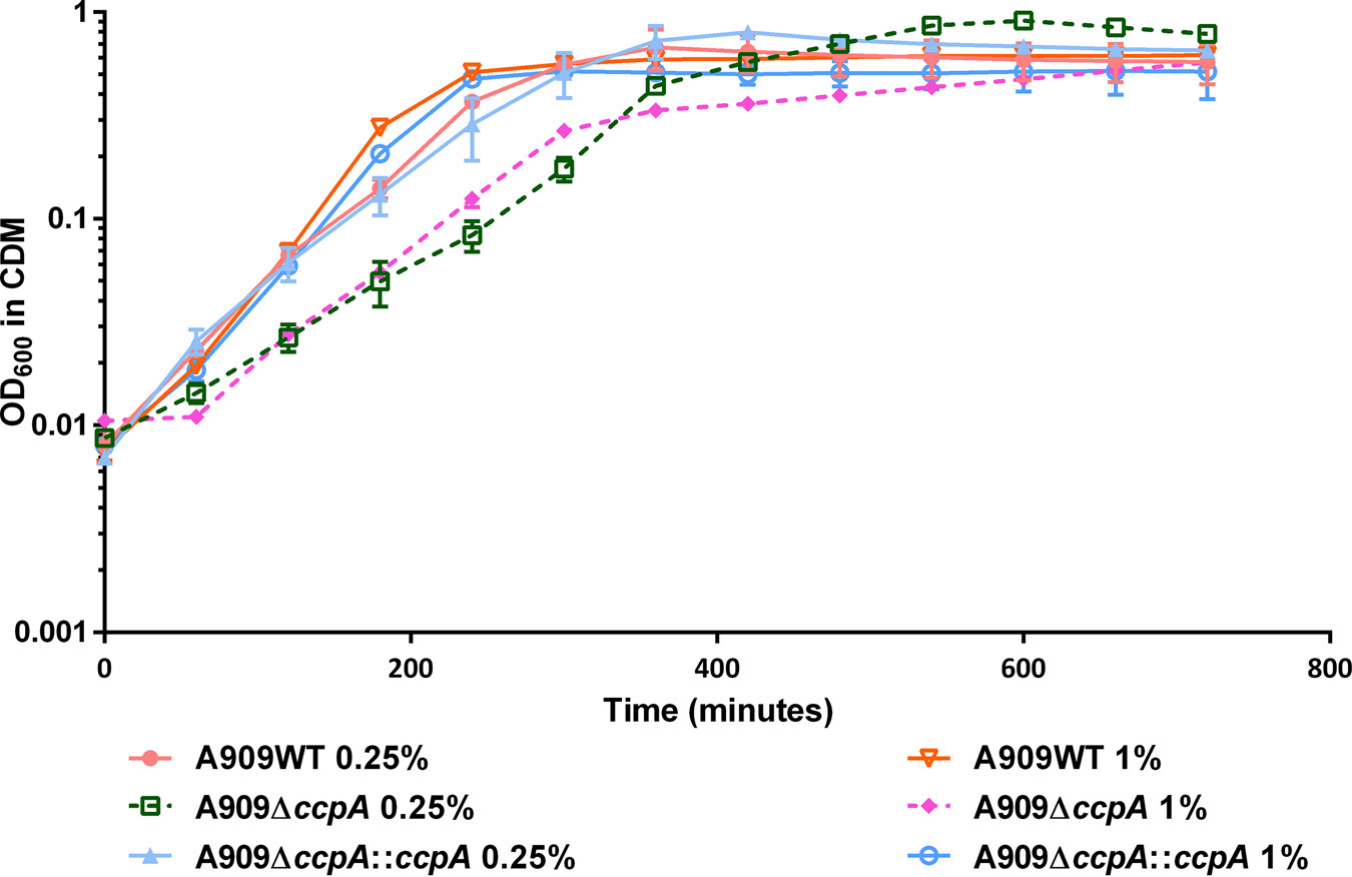
Growth of S. agalactiae strains in chemically defined medium. Growth of A909WT, A909Δ*ccpA*, and A909Δ*ccpA*::*ccpA* in CDM medium supplemented with 0.25% or 1% glucose in a 96-well microplate. The curves are means ± standard deviation (SD) over at least two independent biological replicates with three technical replicates for each.

In order to identify the CcpA regulon and to determine the global impact of glucose on gene expression, we performed transcriptome analysis of the strains A909WT and A909Δ*ccpA* in the mid-exponential phase of growth in a chemically defined medium (CDM) supplemented with glucose. The mutant strain A909Δ*ccpA* exhibited a growth delay which was reversed in the complemented A909Δ*ccpA*::*ccpA* strain (Fig. 1). Hence, we determined that the wild-type and A909Δ*ccpA*::*ccpA* strains reached the mid-exponential phase at an optical density at 600 nm (OD_600_) of 0.45, and the mutant strain A909Δ*ccpA* reached an OD_600_ of 0.3. To determine the appropriate glucose concentrations for the transcriptome, we first performed growth curves with glucose concentrations ranging from 0.1 to 5%. As shown in Fig. S1 in the supplemental material, the A909WT and Δ*ccpA* strains showed major growth defects with 0.1% and 5% glucose. In addition, the stationary phase of the Δ*ccpA* mutant with 2% glucose in the medium had a significantly decreased OD. Thus, for RNA sequencing (RNA-seq) experiments, we decided to supplement the chemically defined medium with 0.25% or 1% glucose, which were both the extreme concentrations without any phenotypic variation in both strains’ growth.

**FIG 2:**
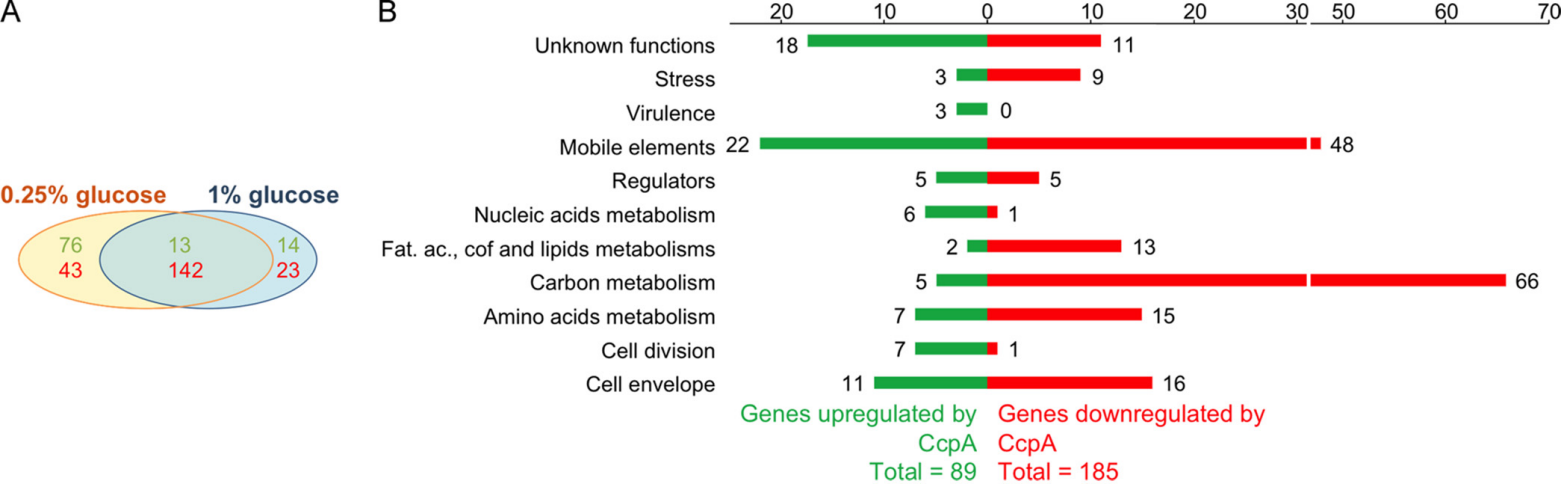
Transcriptome analysis of differentially expressed genes between S. agalactiae A909WT and A909Δ*ccpA* growing in CDM with glucose in the mid-exponential phase. (A) Number of genes differentially expressed in S. agalactiae A909Δ*ccpA* compared to the WT in CDM with 0.25% or 1% glucose. Green, genes upregulated by CcpA; red, genes downregulated by CcpA; black, not regulated by CcpA. (B) Transcriptome analysis of functional classes of differentially expressed genes between A909WT and A909Δ*ccpA* growing in CDM with 0.25% glucose. Pathway predictions and gene annotations were conducted with the Kyoto Encyclopedia of Genes and Genomes (KEGG Pathway) (https://www.kegg.jp/kegg/) (92) and the MicroScope platform (https://www.genoscope.cns.fr/agc/microscope) (90), as explained in Materials and Methods. Experiments were performed over three independent biological replicates. Individual genes from each category are listed in Table S3. Fat. ac, fatty acids; cof, cofactors.

**FIG 3:**
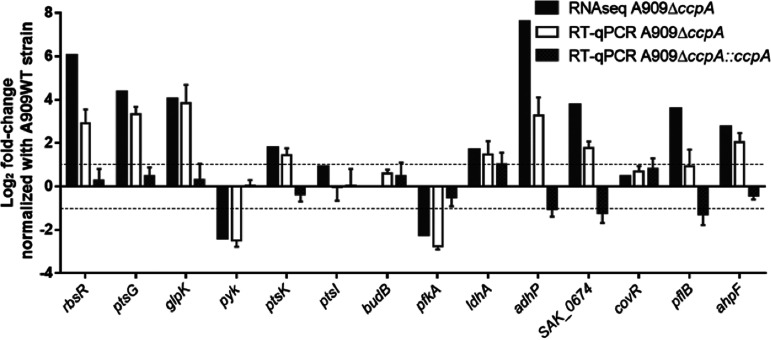
Validation of RNA-seq by RT-qPCR. The transcript levels of 14 genes of the A909WT, A909Δ*ccpA*, and A909Δ*ccpA*::*ccpA* strains were detected by RT-qPCR. The number of transcripts of each gene was normalized against transcript levels of 2 housekeeping genes (*gyrB* and *rpoB*). Gene expression is presented as the log_2_ fold change of the A909Δ*ccpA* mutant or A909Δ*ccpA*::*ccpA* normalized to the A909WT transcript levels. The data are means ± SD of three independent biological replicates with three technical replicates for each. The dotted lines represent a log_2_ fold change of −1 and 1. The 14 selected genes are *rbsR* (*SAK_0171*), *ptsG* (*SAK_1920*), *glpK* (*SAK_0345*), *pyk* (*SAK_1037*), *ptsK* (*SAK_0862*), *ptsI* (*SAK_0946*), *budB* (*SAK_1279*), *pfkA* (*SAK_1036*), *ldhA* (*SAK_0821*), *adhP* (*SAK_0087*), *SAK_0674*, *covR* (*SAK_1639*), *pflB* (*SAK_1735*), and *ahpF* (*SAK_1854*).

Transcriptome analysis revealed no overall effect of glucose concentration between the two tested conditions. Only 7 genes belonging to five transcriptional units were differentially regulated by increasing the glucose concentration from 0.25% to 1% in the A909WT strain: *SAK_0087* and *SAK_1651* coding two alcohol dehydrogenases, *SAK_0170* and *SAK_0171* belonging to a ribose operon, *SAK_0528* and *SAK_0529* belonging to a galactitol PTS operon, and *SAK_0257* coding a trehalose PTS. In order to demonstrate a glucose effect in S. agalactiae, the A909WT strain was cultured in chemically defined medium supplemented with different carbon sources (glucose, fructose, galactose, saccharose, and ribose) alone or together. At the mid-exponential phase of growth, RNAs were extracted and reverse transcriptase quantitative PCR (RT-qPCR) was performed on four genes (*ccpA*, *rbsR*, *pyk*, and *ptsG*) known to be regulated by glucose in other Gram-positive bacteria. Overall, there was no transcriptional difference between all tested conditions, making it impossible to show a glucose effect on the gene regulation of S. agalactiae (Fig. S2) under our conditions. Despite the apparent lack of glucose effect, 274 and 192 genes were differentially regulated by CcpA under 0.25% and 1% glucose, respectively, when comparing the A909Δ*ccpA* mutant and the parental strain (Fig. 2). To simplify the transcriptional analysis, only the results obtained for the 0.25% glucose condition will be presented. The transcriptome analysis revealed that 274 genes, i.e., approximately 150 transcriptional units, among the 2,028 S. agalactiae A909 genes studied, were differentially transcribed, corresponding to 13.5% of the genome. Genes downregulated by CcpA (i.e., upregulated in the A909Δ*ccpA* mutant) outnumbered the upregulated genes (i.e., downregulated genes in the A909Δ*ccpA* mutant) by a factor of two to one. Genes differentially transcribed were mostly genes involved in carbon metabolism (Fig. 2, Table S3). To confirm the data obtained by RNA-seq, RT-qPCRs were performed on 14 genes of different cell functions, whether regulated or not, with RNA prepared from strains A909WT, A909Δ*ccpA*, and A909Δ*ccpA*::*ccpA* under the same conditions as for RNA-seq but with completely independent samples. RT-qPCR results confirmed the RNA-seq data for the tested genes. Furthermore, the level of expression was complemented in strain A909Δ*ccpA*::*ccpA* (Fig. 3).

### The major part of the S. agalactiae CcpA regulon is directly regulated through *cre* sites.

We next searched for the presence of the *cre* motif predicted by RegPrecise, by screening the genome with Virtual Footprint and RegPrecise. With Virtual Footprint, we identified 490 potential *cre* sites corresponding to 418 genes that harbored at least one putative *cre* site. Among these 418 genes, 82 were significantly regulated in the transcriptome analysis, 18 were upregulated by CcpA, and 64 were downregulated by CcpA. Among the 89 genes upregulated by CcpA, as determined by RNA-seq analysis, only 10 had a *cre* site located in a position considered to be activating, i.e., upstream of the −35 box of their transcriptional unit (*SAK_0419*, *SAK_0575-0576*, *SAK_0685*, *SAK_0902*, *SAK_0950*, *SAK_1233-1234*, *SAK_1394*, and *SAK_1640*). Instead, upregulation of most genes by CcpA probably occurs indirectly.

**FIG 4:**
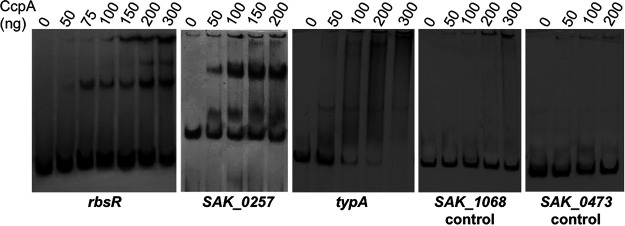
Binding of CcpA to different targets with *cre* sites. EMSAs of DNA fragments containing the *cre* sites of *rbsR* (*SAK_0171*), *SAK_0257*, and *typA* (*SAK_0575*) genes and containing no *cre* site to serve as negative control (*SAK_1068* and *SAK_0473*) were performed. The digoxigenin-labeled DNA fragments were incubated with increasing concentrations of purified CcpA-His6 as described in Materials and Methods.

To confirm the predicted *in silico cre* site, EMSAs were performed using S. agalactiae CcpA and DNA fragments spanning the region containing the putative *cre* site of three genes regulated by CcpA (*rbsR*, *typA*, and *SAK_0257* encoding the trehalose phosphotransferase system) under the transcriptome conditions. Proteins encoded by *rbsR* and *SAK_0257* are both involved in carbon metabolism. The gene *typA* codes for a translational regulator involved in the expression of virulence and pathogenicity factors under carbon starvation in Escherichia coli (45) and cold shock response in B. subtilis (46). Regions belonging to the *SAK_0473* and *SAK_1068* genes without *cre* sites served as negative controls. The addition of CcpA induced a shift in mobility of the three probes containing a *cre* site from 50 ng of CcpA for the three genes (Fig. 4).

### CcpA is largely involved in carbohydrate metabolism.

Among the genes coding the 17 putative PTS predicted in S. agalactiae (KEGG organisms: https://www.genome.jp/kegg/catalog/org_list.html), 7 were inhibited by CcpA through direct interaction with a *cre* site. CcpA inhibited genes coding PTS of rapidly metabolizable sugars such as *ptsG*, coding the enzyme EIIABC of the glucose PTS system, and genes coding PTS of known secondary sugars such as the *SAK_0528-SAK_0530* genes, encoding a potential galactitol PTS transporter carried by the insertion sequence IS*1381*. Three operons encoding ABC transporters involved in carbon metabolism were downregulated by CcpA: *SAK_0532-0539*, *SAK_0166*-*SAK_0171*, and *SAK_1475*-*SAK_1477* coding the transporters of *N*-acetylglucosamine, ribose, and cyclodextrin, respectively, for which a *cre* site was present.

**FIG 5:**
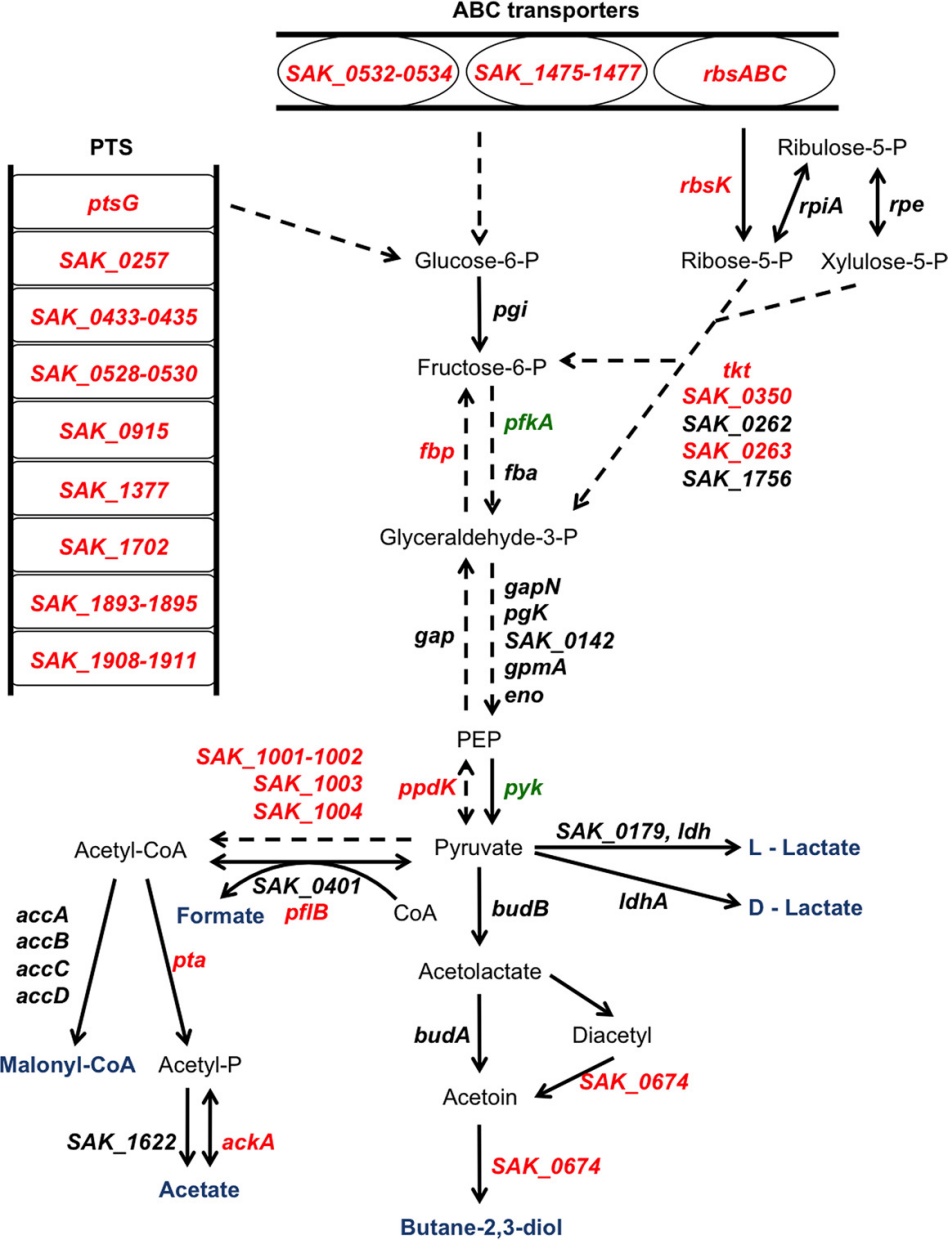
Schematic model of the regulon of CcpA in carbohydrate utilization, central carbon metabolism, and fermentation of S. agalactiae. Schematic overview of the role of CcpA in regulating carbohydrate utilization, central carbon metabolism and fermentation in S. agalactiae as derived from gene expression in the RNA-seq. Green, gene upregulated by CcpA; red, gene downregulated by CcpA; black, unregulated by CcpA. Solid arrows represent direct reactions, and dashed arrows represent reactions for which there are intermediate reactions. Pathway predictions and gene annotations were conducted with the Kyoto Encyclopedia of Genes and Genomes (KEGG Pathway) (https://www.kegg.jp/kegg/) (92) and the MicroScope platform (https://www.genoscope.cns.fr/agc/microscope) (90) as explained in Materials and Methods. *ptsG* (*SAK_1920*), PTS system, glucose-specific IIABC component; *SAK_0257*, PTS system, trehalose-specific IIABC component; *SAK_0398-0400*, lactose/cellobiose PTS, IIA, IIB, and IIC subunits; mannose/fructose/sorbose PTS, IID, IIC, IIB, and IIA subunits; *SAK_0433-0435*, PTS system, IID component, mannose/fructose/sorbose family; *SAK_0528-0530*, galactitol PTS, IIA, IIC, and IIB subunits; *SAK_0915*, PTS system, β-glucoside-specific IIABC component; *SAK_1377*, PTS system, fructose-specific IIABC component; *SAK_1702*, PTS system, sucrose-specific IIABC component; *SAK_1893-1895*, PTS system, glucose-specific IIABC component; *SAK_1908-1911*, mannose/fructose/sorbose PTS, IID, IIC, IIB, and IIA subunits, *SAK_0532-0534*, sugar ABC transporter; *SAK_1475-1477*, cyclodextrin ABC transporter; *rbsABC* (*SAK_0166-0168*), ribose ABC transporter; *rbsK* (*SAK_0170*), ribokinase; *rpiA* (*SAK_1270*), ribose 5-phosphate isomerase A; *rpe* (*SAK_1798*), ribulose-phosphate 3-epimerase; *tkt* (*SAK_0350*), transketolase; *SAK_0262*,*0263*, putative transketolase, N- and C-terminal subunits; *SAK_1756*, transketolase; *pgi* (*SAK_0475*), glucose-6-phosphate isomerase; *fbp* (*SAK_0666*), fructose-1,6-biphosphatase; *pfkA* (*SAK_1036*), 6-phosphofructokinase; *fbA* (*SAK_0178*), fructose-1,6- biphosphate aldolase; *gapN* (*SAK_0947*), glyceraldehyde-3-phosphate dehydrogenase; gap (*SAK_1790*), glyceraldehyde-3-phosphate dehydrogenase; *pgk* (*SAK_1788*), phosphoglycerate kinase; *SAK_0142*, phosphoglycerate mutase; *gpmA* (*SAK_0889*), phosphoglyceromutase; *eno* (*SAK_0713*), phosphopyruvate hydratase; *pyk* (*SAK_1037*), pyruvate kinase; *ppdK* (*SAK_1682*), pyruvate phosphate dikinase; *SAK_0179*, L-2-hydroxyisocaproate dehydrogenase; *ldh* (*SAK_1054*), l-lactate dehydrogenase; *ldhA* (*SAK_0821*), d-lactate deshydrogenase; *budB* (*SAK_1279*), acetolactate synthase; *budA* (*SAK_1278*), alpha-acetolactate decarboxylase; *SAK_0674*, acetoin reductase; *pta* (*SAK_1177*), phosphotransacetylase; *SAK_1622*, acylphosphatase; *ackA* (*SAK_0234*), acetate kinase; *SAK_1001-1002*, *SAK_1003*, *SAK_1004* acetoin dehydrogenase; *SAK_0401*, formate acetyltransferase 2; *pflB* (*SAK_1735*), formate acetyltransferase 1; *accA*, *accBCD* (*SAK_0424*, *SAK_0426-0428*) acetyl-CoA carboxylase.

Several genes involved in glycolysis and gluconeogenesis were regulated by CcpA, as already demonstrated in other *Firmicutes* (32, 40, 47). CcpA upregulated *pfkA*, which codes the PfkA protein, a key enzyme of glycolysis, catalyzing the conversion of fructose-6-phosphate to fructose-1,6-biphosphate. Conversely, the gene coding the enzyme catalyzing the opposite reaction, fructose-1,6-biphosphatase, Fbp, was downregulated by CcpA and harbored a *cre* site. Likewise, the *pyk* gene was upregulated by CcpA, whereas the *ppdK* gene was downregulated by CcpA (Fig. 5, Table S3).

S. agalactiae being a bacterium involving a fermentative metabolism of sugars, such as glucose, we were interested in the role of CcpA in the fermentation process. Thereby, five main glucose fermentation pathways are identified in S. agalactiae; three of them were partially or totally regulated by CcpA. The main mode of S. agalactiae fermentation involves the action of lactate dehydrogenases, which transform the pyruvate produced by glycolysis into lactate. Three genes encoding lactate dehydrogenases (*ldh*, *SAK_0179*, and *ldhA*) have been identified in S. agalactiae, but none of them was differentially expressed in the A909Δ*ccpA* strain under the test conditions. Thus, although most of the pyruvate is converted to lactic acid in S. agalactiae, CcpA was not involved in the regulation of lactate dehydrogenases under our conditions. The second identified fermentation pathway results in the formation of butane-2,3-diol. Among the different enzymes involved in this fermentation pathway, only acetoin reductase, encoded by the gene *SAK_0674*, was significantly inhibited by CcpA in the presence of glucose. This gene has a *cre* site. The third fermentation pathway is characterized by the production of acetate from pyruvate derived from glycolysis via the acetyl phosphate intermediate. Phosphotransacetylase and acetate kinase, respectively, encoded by the *pta* and *ackA* genes that possess *cre* sites, were downregulated by CcpA. Finally, glucose fermentation in S. agalactiae can also lead to the production of formate. The transcriptomic analysis revealed that the genes *SAK_1001-1002*, *SAK_1003*, and *SAK_1004*, encoding the enzymes involved in the formation of formate from pyruvate, were downregulated by CcpA with the gene *SAK_1001* presenting a *cre* site (Fig. 5).

To confirm the impact of CcpA regulation on several fermentation processes, we analyzed by nuclear magnetic resonance (NMR) spectroscopy the end products of fermentation in the culture supernatants of strain A909WT, the *ccpA* mutant and its complemented strain grown in chemically defined medium supplemented with 0.25% glucose. We could not detect butane-2,3-diol or malonyl-CoA with these assays. For technical reasons, lactate and threonine were measured together. There were no significant differences in the lactate plus threonine concentration between the three strains. Conversely, there was an overproduction of acetate (fold change of 4.14 compared to strain A909WT) and formate (fold change of 1.87 compared to strain A909WT) by the *ccpA* mutant (Fig. S3). These results were in agreement with our transcriptome data since CcpA does not regulate lactate dehydrogenases but represses genes involved in acetate and formate production.

### CcpA also largely regulates the expression of mobile genetic elements.

Genes belonging to three mobile genetic elements were regulated by CcpA: the LambdaSa03 prophage found in S. agalactiae strains A909, H36B, and 515, the LambdaSa04 prophage found in S. agalactiae strains A909 and CJB111 (48), and the genetic mobile element containing the insertion sequence IS*1381*. In particular, a large part of the genes of the LambdaSa03 and LambdaSa04 prophages, mostly with unknown function, were significantly upregulated (22 out of 49 genes) or downregulated (37 out of 48 genes), respectively. Out of 20 genes from a mobile genetic element containing the IS*1381*, 11 were downregulated by CcpA and code proteins involved in carbon metabolism.

### CcpA influences the stress response.

**FIG 6:**
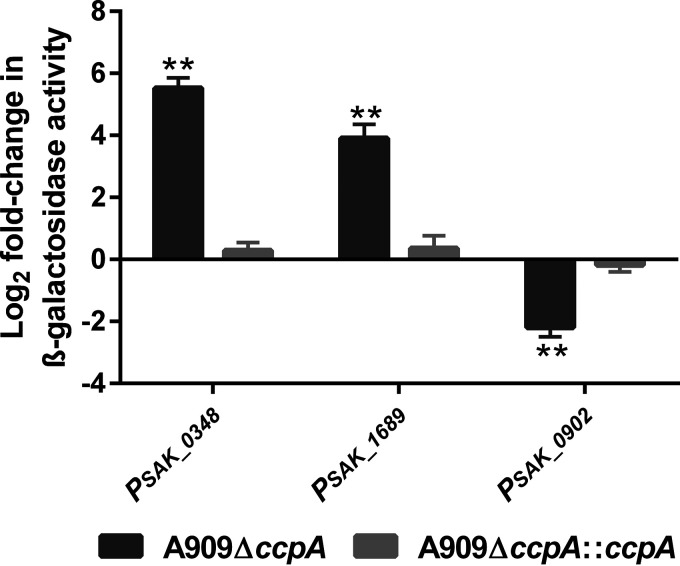
CcpA regulates genes putatively involved in the stress response. A909 WT, its isogenic mutant A909Δ*ccpA*, and the strain complemented *in situ*, A909Δ*ccpA*::*ccpA*, were transformed with pTCV-*lacZ* containing the promoter regions of *SAK_0348*, *SAK_1689*, and *SAK_0902* upstream the *lacZ* gene (P*_SAK_0348_*, P*_SAK_1689_*, and P*_SAK_0902_*, respectively). A909 cells were grown in CDM supplemented with 0.25% glucose until they reached the mid-exponential phase. β-Galactosidase assays were performed as described in Materials and Methods. Promoter activity of the strains is expressed as the log_2_ fold change relative to the WT strain. The values shown are presented as the means ± SD over four independent biological experiments. The asterisks indicate *P* values obtained using one-sample *t* test. **, *P* < 0.01.

Interestingly, a total of 12 genes belonging to 9 transcriptional units and putatively involved in the stress response were regulated by CcpA (Table S3). Among them, eight had a *cre* site in their regulatory region. In addition, four other genes involved in stress responses (*lytR*, *ciaR*, and *ahpC-F*) and regulated by CcpA were located elsewhere in the transcriptome because of their other physiological functions. For instance, *cidA-B* (*SAK_1233-1234*) was upregulated; *lrgA-B* (*SAK_0250-0251*) and *lytR* (*SAK_0247-0249* operon), which code a two-component system transcriptional regulator known to regulate *lrgA-B*, were downregulated by CcpA thanks to the presence of a *cre* site in the regulatory region of each operon. These genes have already been shown to affect stress responses and pathogenicity in S. mutans (27, 49–51). We confirmed the regulation by CcpA for three of the genes presenting a *cre* site thanks to transcriptional fusions: *SAK_0348*, an NADH peroxidase previously reported to play a role in oxidative stress (52); *SAK_0902*, coding a putative DEAD box ATP-dependent RNA helicase, which, by sequence analogy (60% identity) with Listeria monocytogenes Lmo0866, could play a role in ethanol stress resistance (53); and *SAK_1689*, coding a putative UspA protein whose regulation in stress is discussed below (Fig. 6).

### CcpA is involved in acid and oxidative stress responses.

**FIG 7:**
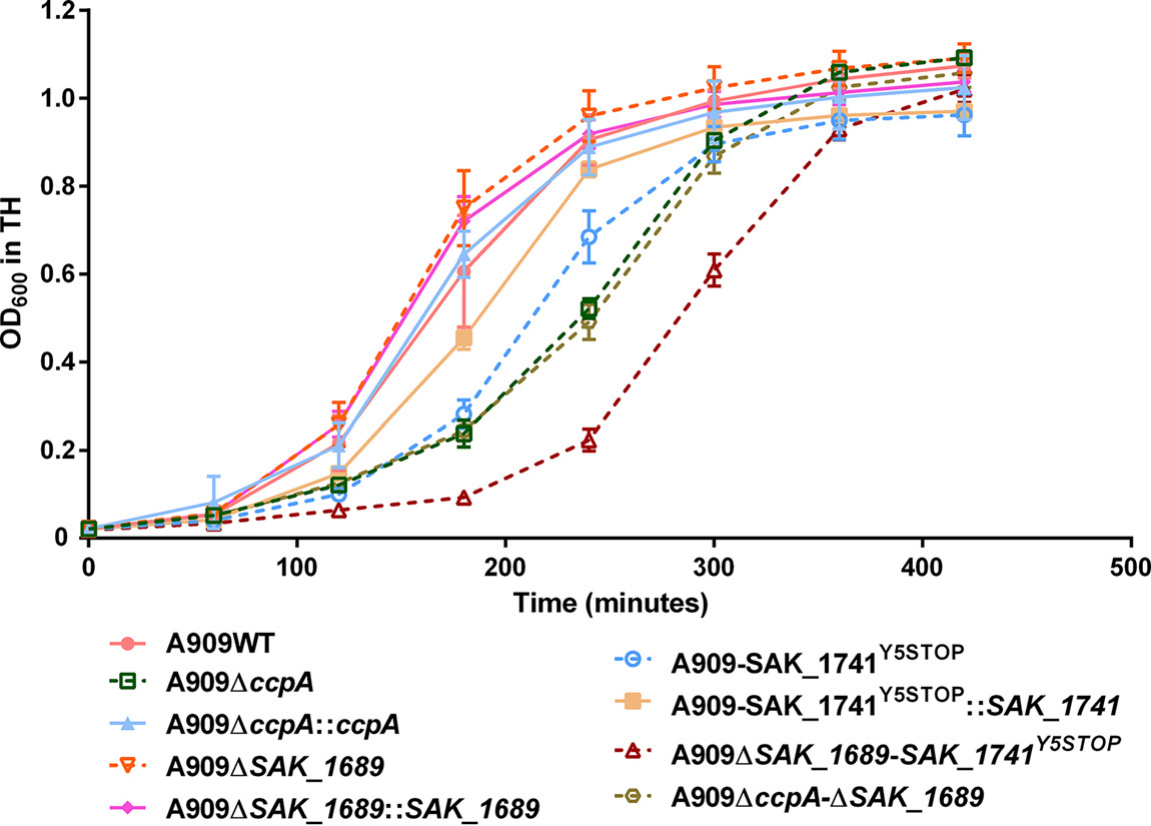
Growth of S. agalactiae strains in TH medium. Growth of A909WT, A909Δ*ccpA*, A909Δ*ccpA*::*ccpA*, A909Δ*SAK_1689*, A909Δ*SAK_1689*::*SAK_1689*, A909-SAK_1741^Y5STOP^, A909-SAK_1741*^Y5STOP^*::*SAK_1741*, A909Δ*SAK_1689*-SAK_1741^Y5STOP^, and A909Δ*ccpA* Δ*SAK_1689* in TH medium in a 96-well microplate. The curves are means ± SD over three independent biological replicates with three technical replicates for each.

**FIG 8:**
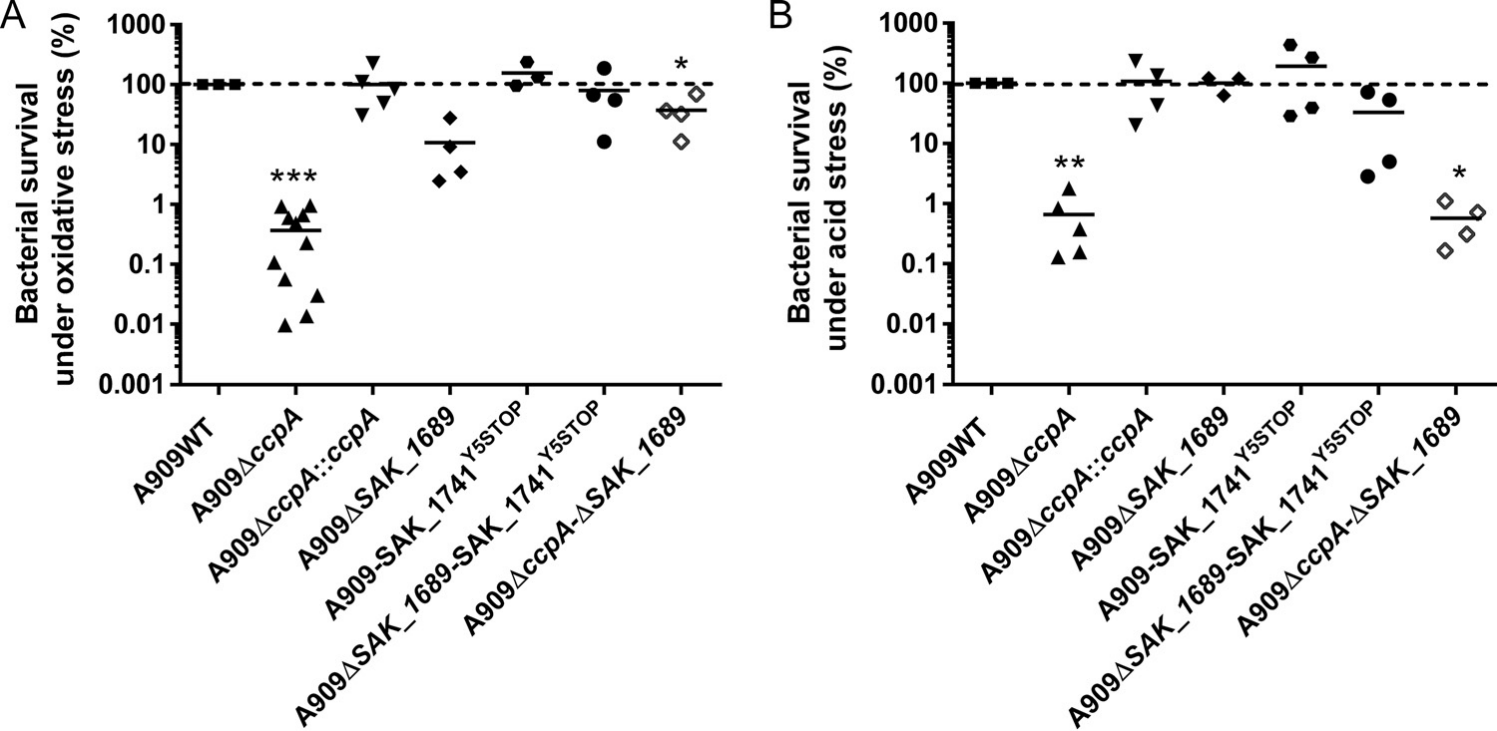
Role of CcpA and SAK_1689 in stress survival. (A) Sensitivity of S. agalactiae to H_2_O_2_. Strains were treated with 20 mM H_2_O_2_ for 30 min. (B) Sensitivity of S. agalactiae to acidic pH (pH 4) after 24 h as described in Materials and Methods. All strains were grown until the mid-exponential phase and then exposed to stresses. Viability was determined by plating onto TH agar. The data are presented as the percent survival relative to a theorical value of 100% for the A909WT strain. Experiments were performed at least three times; each point represents an independent biological experiment. The asterisks indicate *P* values obtained using a Wilcoxon test, comparing the WT strain CFU to those of the other tested strains. *, *P < *0.05; **, *P < *0.01; ***, *P < *0.001.

After the growth of strains A909WT, A909Δ*ccpA*, and A909Δ*ccpA*::*ccpA* in Todd Hewitt (TH) broth (Fig. 7), until mid-expential phase, we performed survival experiments in TH medium containing 20 mM H_2_O_2_ or in acidic TH (pH = 4) to assess the role of CcpA in stress conditions. We observed that the percent survival of strain A909Δ*ccpA* was impaired 270- and 152-fold under these two conditions, respectively, compared to the wild-type strain (Fig. 8A and B). These results indicate that CcpA plays an essential role in S. agalactiae in conferring protection against oxidative and acidic stresses (Fig. 8).

### SAK_1689 codes a putative UspA protein and is overexpressed under oxidative stress.

To explain the susceptibility of the A909Δ*ccpA* mutant strain, we searched for its direct targets putatively involved in stress responses and regulated in our transcriptomic analysis. Our transcriptome analysis indicates that *SAK_1689* was downregulated by CcpA with a log_2_ fold change of 4.91 (Table S3). To the best of our knowledge, the function of the resulting protein has not yet been characterized in S. agalactiae. This gene presents a *cre* site which spans the −35 box. It codes a putative universal stress protein A (UspA) with 52% sequence identity and 70% sequence similarity with Lmo1580 of Listeria monocytogenes EGDe (Fig. S4). The latter is involved in acidic and oxidative stresses and survival in macrophages (54). The role of UspA proteins remains unclear, but they might play a role in DNA protection or in posttranslational polypeptide modifications in E. coli (55, 56). One study has already demonstrated that the putative UspA protein coded by *SAK_1689* is overexpressed under nutrient stress (extended stationary phase) in S. agalactiae (57).

**FIG 9:**
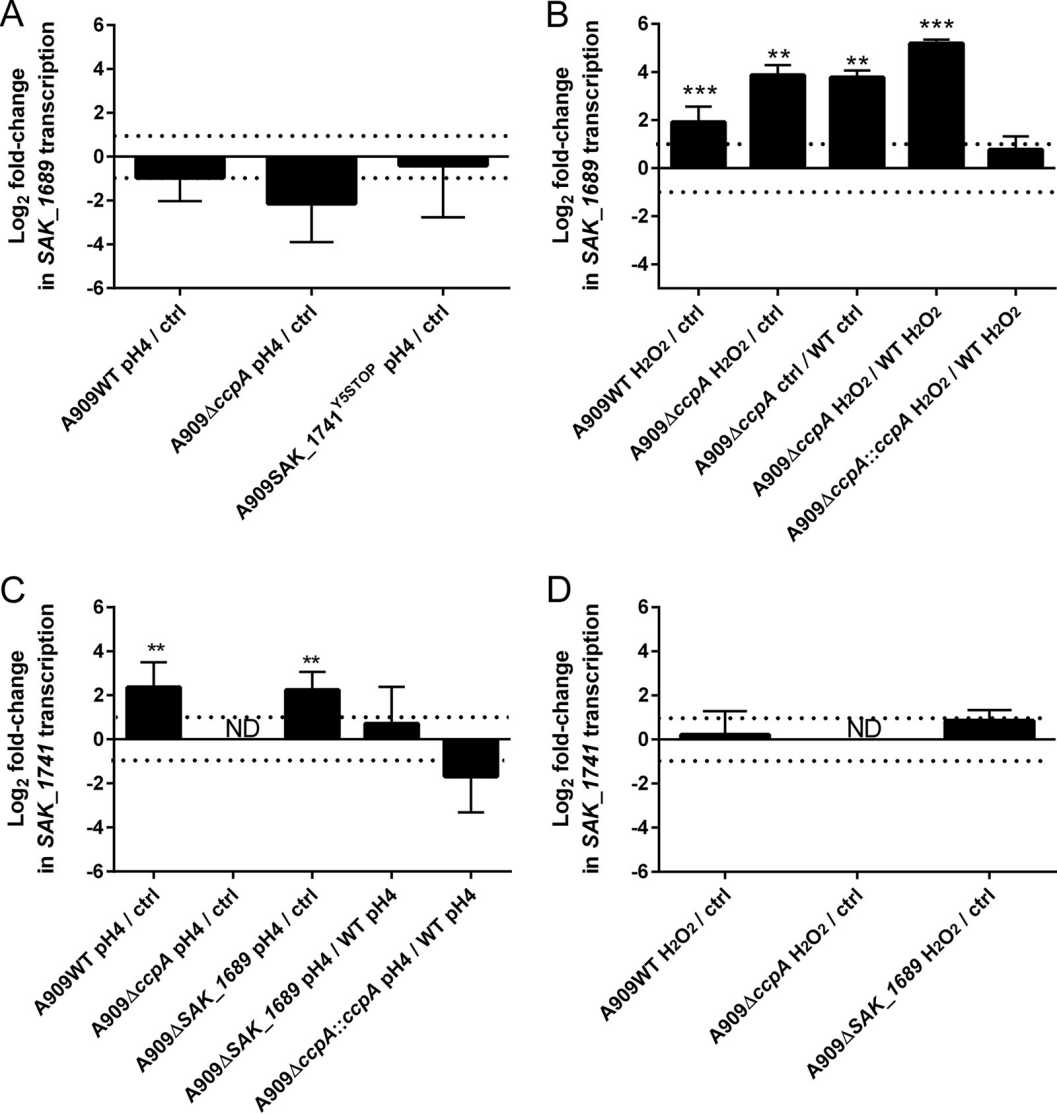
Transcription of *SAK_1689* and *SAK_1741* under stress conditions. qRT-PCRs were performed on RNA extracts of S. agalactiae strains grown to the mid-exponential phase. The number of transcripts of each gene was normalized against *recA* transcript levels. Gene expression is presented as the log_2_ fold change in *SAK_1689* and *SAK_1741* transcript levels. Ctrl stands for control condition, i.e., for each strain, the condition without stress in TH at pH 7. ND means expression not detected (>30 threshold cycle [*C_T_*]). The dotted lines represent a log_2_ fold change of −1 and 1. (A and C) Transcription of *SAK_1689* (A) and *SAK_1741* (C) under acidic conditions was measured after 20 min of stress in TH at pH 4. (B and D) Transcription of *SAK_1689* (B) and *SAK_1741* (D) under oxidative conditions was measured in TH after 20 min of stress in 2.5 mM H_2_O_2_ (MIC). Results are presented as the means ± SD over at least three independent biological replicates with three technical replicates for each. *P* values were obtained using a one-sample *t* test to compare the log_2_ fold change to a hypothetical value of 0 (no difference). **, *P < *0.01; ***, *P < *0.001.

The expression of *SAK_1689* in acidic TH (pH 4) or TH containing 2.5 mM hydrogen peroxide (=MIC) was evaluated by RT-qPCR. No overexpression was observed in acidic pH (Fig. 9A), though we showed an overexpression of the gene under oxidative conditions (log_2_ fold change of 1.91) in the wild-type strain (Fig. 9B). To determine whether CcpA was involved in the regulation of *SAK_1689* under oxidative conditions, we performed RT-qPCR with strain A909Δ*ccpA* in TH containing 2.5 mM hydrogen peroxide. The gene was upregulated under oxidative conditions with a log_2_ fold change of 3.96 (Fig. 9B). The expression of *SAK_1689* in the A909Δ*ccpA* strain compared to the A909WT strain under oxidative conditions was significantly higher (log_2_ fold change of 5.19). Thus, *SAK_1689* was significantly more transcribed in the Δ*ccpA* mutant than in the wild-type (WT) strain under oxidative conditions. Therefore, we hypothesize that an unknown activator upregulates *SAK_1689* under oxidative conditions and that CcpA limits this overexpression.

### SAK_1689 and SAK_1741 Usp proteins are not essential for stress resistance in S. agalactiae.

Since *SAK_1689* is overexpressed under oxidative stress, a deletion mutant and its complemented strain were first constructed. After growth in the TH medium (Fig. 7), survival experiments under oxidative stress were performed as for the mutant A909Δ*ccpA* to determine the role of *SAK_1689*. Under oxidative stress, we observed a lower survival rate of the A909Δ*SAK_1689* mutant that was not statistically significant (*P* value of 0.057) (Fig. 8A). However, the survival experiments under acidic conditions did not show any impaired survival rate for the A909Δ*SAK_1689* mutant (Fig. 8B). To explain the absence of these significant phenotypes with the A909Δ*SAK_1689* strain, we hypothesized that there might be functional redundancy between SAK_1689 and other protein(s) under stress conditions. Usp-containing organisms are, indeed, usually equipped with several *usp* genes. Interestingly, *in silico* analysis of the S. agalactiae genome permits the detection of a paralog of *SAK_1689*, *SAK_1741*, encoding a protein that has 67% sequence identity with SAK_1689 (Fig. S4). The *SAK_1741* gene is not significantly regulated by CcpA, nor does it have *cre* site. Thus, we hypothesized that there might be functional redundancy between SAK_1689 and SAK_1741 under stress conditions. An RT-qPCR experiment that analyzed *SAK_1741* expression in A909WT, A909Δ*ccpA*, and A909*ΔSAK_1689* strains showed that this gene was weakly expressed in all the strains, particularly in A909Δ*ccpA*. No difference in *SAK_1741* gene expression was found, except under acidic stress conditions, where the transcription of *SAK_1741* was higher in the WT and A909Δ*SAK_1689* strains (log_2_ fold change of 2.37 and 2.24, respectively) (Fig. 9C and D). We therefore generated the A909-SAK_1741^Y5STOP^ substitution mutant and the A909*ΔSAK_1689-*SAK_1741^Y5STOP^ double mutant to evaluate their survival under stress conditions and to determine whether these two Usp proteins were (i) involved in stress responses and (ii) functionally redundant even if the expression of *SAK_1741* was not differentially affected under stress conditions in the Δ*SAK_1689* mutant. The growth of the three strains was first determined in TH medium and highlighted a growth delay for A909-SAK_1741^Y5STOP^ and A909*ΔSAK_1689* SAK_1741^Y5STOP^ strains (Fig. 7). However, like for the A909Δ*SAK_1689* mutant, the survival under oxidative or acidic conditions of the A909-SAK_1741^Y5STOP^ mutant and the double A909*ΔSAK_1689* SAK_1741^Y5STOP^ mutant was not significantly impacted (Fig. 8A and B), demonstrating that the phenotype observed for A909Δ*SAK_1689* under stress was not due to a compensatory effect.

### Overproduction of SAK_1689 in the A909Δ*ccpA* mutant under oxidative conditions is harmful.

We hypothesized that CcpA represses *SAK_1689* under the oxidative stress conditions and prevents the overproduction of SAK_1689 that could be harmful to the bacteria. Indeed, in E. coli, Nyström et al. (55) showed that overproduction of UspA results in a significant reduction in cell growth rate in minimal medium, along with alterations in global protein synthesis and modifications of the pI of some proteins. Thus, the double deletion mutant A909Δ*ccpA* Δ*SAK_1689* was constructed, and after growth in TH medium (Fig. 7), survival experiments under oxidative stress were performed as mentioned before. We observed that the percentage of survival was 2.7-fold lower than that of the wild-type strain but 109.6-fold higher than that of the A909Δ*ccpA* strain, thus confirming our hypothesis (Fig. 8A). We can therefore conclude that the impacted survival of the A909Δ*ccpA* mutant under oxidative stress is primarily due to the overexpression of *SAK_1689*. Conversely, as expected, under acid conditions, where the gene *SAK_1689* is not overexpressed, we observed similar survival rates between the A909Δ*ccpA*Δ*SAK_1689* and A909Δ*ccpA* mutants (Fig. 8B). Thus, the impacted survival of the A909Δ*ccpA* mutant under acidic stress is due not to SAK_1689 but probably to other genes regulated by CcpA.

### Importance of the *ccpA* gene for survival in macrophages.

**FIG 10:**
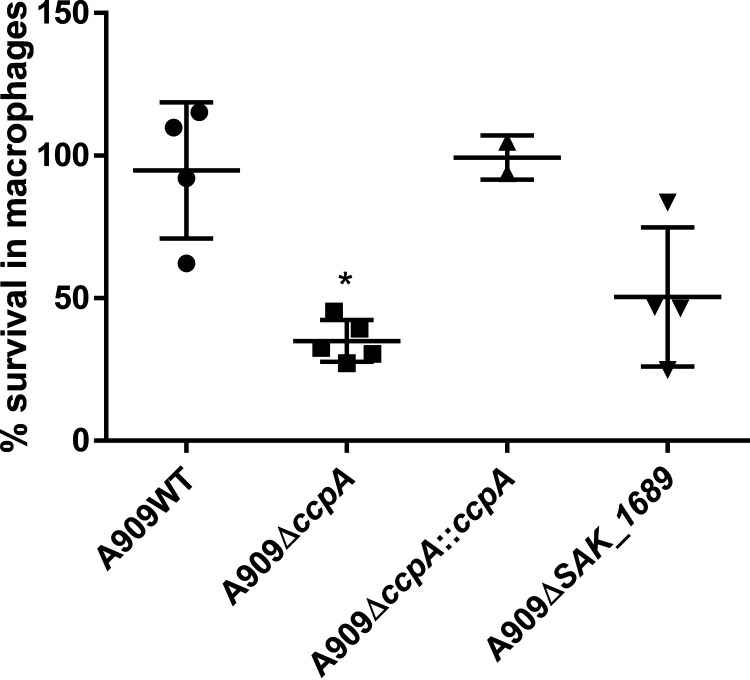
Importance of CcpA for survival in macrophages. Survival of S. agalactiae strains inside RAW 264.7 macrophages was evaluated after 24 h of infection. The number of intracellular bacteria was normalized to the number of bacteria in the well after the antibiotic treatment as described in Materials and Methods. Strains were grown until the mid-exponential phase, and then RAW 264.7 macrophages were infected. Viability was determined by plating on TH agar. Assays were performed over four technical replicates and repeated over at least two independent biological replicates. Each dot represents the mean of technical replicates for one biological experiment. The asterisks indicate *P* values obtained using ANOVA and then Wilcoxon tests. *, *P < *0.05.

We also tested the ability of strains A909WT, A909Δ*ccpA*, A909Δ*ccpA*::*ccpA*, and *A909ΔSAK_1689* to survive inside RAW264.7 macrophage cell lines in order to determine the role of CcpA and SAK_1689 in bacterial survival inside the macrophages. The percentage of survival of the A909Δ*ccpA* mutant after 24 h was 2.7-fold lower than that of the A909WT strain, with a *P* value of 0.01. The controls performed showed that gentamicin treatment killed extracellular bacteria and that RAW 264.7 cells were not killed during the experiment (Fig. S5). The survival of the A909Δ*SAK_1689* mutant was weakly but not significantly impaired (fold change of 1.88) (Fig. 10). Thus, CcpA plays an important role for survival in macrophages, probably due to the regulation of some genes encoding proteins involved in this phenomenon.

## DISCUSSION

A better understanding of the mechanisms involved in the physiology of S. agalactiae is essential to understand how this bacterium adjusts to changes in its environment. The variability of the sites colonized by S. agalactiae attests to its great capacity for adaptation: the ability to acquire nutrients and but also to cope with physicochemical variations such as an acidic pH or oxidative stress in the vagina.

In this study, we deleted the *ccpA* gene in strain A909 to create a *ccpA* mutant which displayed a growth delay. However, Hooven et al., showed that *ccpA* was an essential gene using a transposon insertion sequencing (Tn-seq) system (44). We used TH medium instead of tryptic soy medium to grow the mutant strain, which could be the reason for the discrepancy in results. Furthermore, because of the *en masse* selection of mutants in the Tn-seq system, mutants with low fitness may disappear (58). We showed here that CcpA is a pleiotropic regulator of S. agalactiae since it regulates 13.5% of the genome. This is consistent with what has been shown before for other *Firmicutes* (31–34). Moreover, as for other Gram-positive bacteria, CcpA acts primarily as a repressor and mainly regulates genes involved in carbon metabolism (31–34, 59) by binding to a *cre* site whose sequence has been validated in our study. In addition to the role of CcpA in carbon metabolism, studies have highlighted the involvement of CcpA in many other functions in other bacteria: in amino acid metabolism (60–64), phosphorus metabolism (65), stress response (32, 65), sporulation (32), colonization (66), biofilm formation (67), and virulence (32, 40, 68, 69). In this study, transcriptome analysis showed that CcpA regulates several genes involved or putatively involved in the stress response. Among the targets of CcpA, we noticed the *SAK_0348* (or *npx*) gene highly downregulated by CcpA, which presents a *cre* site and has been previously shown to be important in an *in vitro* model of multiple phagosomal biochemical/oxidant stressors or in macrophages (52). Further, CcpA downregulates two peroxidases: a thiol peroxidase (Tpx) without a *cre* site and an alkyl hydroperoxide reductase (AhpCF), whose role was described by Lechardeur et al. (26) and that presents a *cre* site. Moreover, as for S. mutans, the genes *lrgA-B* and *lytR* downregulated by CcpA and *cidA-B* upregulated by CcpA could play a role in oxidative stress resistance in S. agalactiae (27, 49–51). This led us to subject strain A909Δ*ccpA* to different types of stresses encountered in the host, such as acidic and oxidative stresses and survival in macrophages. As the survival of the mutant A909Δ*ccpA* was highly impacted under these conditions, we concluded that CcpA was crucial in stress resistance. Although several transcriptome studies have shown that CcpA regulates genes involved in the stress response (32, 65), very few phenotypic studies have been performed to show the involvement of CcpA in stress resistance. Two studies in *Lactobacillus* species showed that, contrary to what we observed, deletion of CcpA affected resistance under several stresses but improved resistance to oxidative stress (70, 71).

Endogenous metabolism can produce H_2_O_2_, depending on the substrate used as an energy source (72, 73). In Streptococcus pyogenes, Kietzman et al. showed that hydrogen peroxide production by lactate oxidase LctO was both glucose- and growth phase-regulated through CcpA (74). Like other streptococci, S. agalactiae presumably releases hydrogen peroxide (75). In conclusion, if CcpA is involved in the metabolism and production of hydrogen peroxide, we showed here that it is also involved in the resistance to oxidative stress.

Furthermore, we highlighted here an overexpression of *SAK_1689* under oxidative stress conditions and high repression of *SAK_1689* by CcpA. In L. monocytogenes, it has been suggested that σ^B^ is the activator of the gene under different stress conditions (54). However, this transcription factor does not exist in S. agalactiae (28). Thus, the activator of *SAK_1689* remains to be determined.

In this study, we showed that CcpA is involved in acidic and oxidative stress resistance and protects S. agalactiae from the deleterious effects of overproduction of the SAK_1689 protein under oxidative stress. To our knowledge, this is the first study which highlights the broad involvement of CcpA in the regulation of mobile genetic elements. Thus, we demonstrated that CcpA is a pleiotropic regulator which is involved in stress resistance in Streptococcus agalactiae.

## MATERIALS AND METHODS

### Bacterial strains and growth conditions.

The reference wild-type (WT) S. agalactiae strain used in this study was strain A909, a sequence type 7 (ST7), clonal complex 7 (CC7), serotype IA clinical isolate from a human case of bacteremia. All strains used in this study are listed in Table S1. Escherichia coli strains were grown in Lysogeny broth (LB) medium (MP; catalog [cat.] no. 3002022) at 37°C with agitation or on an LB agar plate. S. agalactiae strains were routinely grown in Todd Hewitt (TH) broth (BD; cat. no. 249240) at 37°C without agitation or on TH-agar plates. When necessary, strains were grown in filter-sterilized chemically defined medium (CDM) (76) supplemented with corresponding carbohydrates: glucose or other sugars.

### Chromosomal and plasmid DNA purification.

Chromosomal DNA of S. agalactiae statically cultured overnight in TH broth was purified by the phenol-chloroform method (77). E. coli plasmids were purified with a NucleoSpin plasmid kit (Macherey-Nagel) according to the manufacturer’s instructions.

### PCR and DNA sequencing.

PCRs were performed with a SimpliAmp thermal cycler (Thermo Fisher Scientific) using Platinum SuperFi high-fidelity DNA polymerase (Thermo Fisher Scientific). The resulting PCR fragments were purified with a NucleoSpin gel and PCR cleanup kit (Macherey-Nagel) according to the manufacturer’s instructions.

PCR products purified with the NucleoSEQ kit (Macherey-Nagel) were sequenced on both strands using the BigDye Terminator (version 3.1) cycle sequencing kit from Applied Biosystems and the ABI Prism 310 genetic analyzer.

### Construction of mutants and complemented strains.

S. agalactiae strain A909WT (78) was used for this study. Its isogenic mutants A909*ΔccpA*, A909*ΔSAK_1689*, and the double mutant A909Δ*ccpA* Δ*SAK_1689* were obtained by allelic exchange thanks to the temperature-sensitive shuttle plasmid pG+host1^ts^ (79). Briefly, upstream and downstream regions of the genes to be deleted were amplified with primers 65F-66R and 67F-68R for A909*ΔccpA* and primers 602F-601R and 600F-599R for A909*ΔSAK_1689*. A recombination cassette, consisting of a fusion between these two regions, was obtained using splicing-by-overlap-extension PCR and cloned into the EcoRI/BamHI restriction sites of the temperature-sensitive shuttle plasmid. Then, the recombinant plasmid was electroporated into an E. coli XL1-Blue strain, plated onto LB agar plates supplemented with 150 μg/mL erythromycin, and incubated overnight at 37°C. The transformants were used for amplification, purification, sequencing, and then electroporation in S. agalactiae strain A909. The transformants were plated on Trypticase soy agar plates with 5% sheep blood supplemented with 10 μg/mL erythromycin at 30°C. Then a liquid culture of the transformant in TH medium with 10 μg/mL erythromycin at 38°C favored a first crossing over. Successive subcultures in TH liquid medium without antibiotics and at 30°C then promoted the second recombination event. Erythromycin-sensitive clones for which a deletion was observed after PCR amplification were sequenced. For the A909*ΔccpA* and A909*ΔSAK_1689* mutants, each deletion mutation is an in-frame, markerless deletion of 984 bp and 432 bp, respectively. To obtain the A909Δ*ccpA* Δ*SAK_1689* mutant, a deletion of the *SAK_1689* gene was performed as explained above in the A909*ΔccpA* strain. To complement these mutants, the entire coding sequence of the genes was amplified by PCR with primers 65F and 68R for A909Δ*SAK_1689* and primers 602F and 599R for A909Δ*SAK_1689* and inserted into pG+host1^ts^, as described above. After allelic exchange, the *in situ* complementation was confirmed by PCR and sequencing.

For the A909-SAK_1741^Y5STOP^ and A909*ΔSAK_1689* SAK_1741^Y5STOP^ mutants, we substituted the 5th codon encoding a tyrosine with a STOP codon. The upstream region of the gene was amplified with one portion of the gene with the primers 593F and 616R, and the downstream region of the gene was amplified with the other portion of the gene with the primers 617F and 618R. The overlap primers 616R and 617F contained the STOP codon instead of the tyrosine to make the substitution. The steps to obtain the mutant were then the same as for a deletion mutant. To complement these mutants, the entire native coding sequence of the *SAK_1741* gene was amplified by PCR with the 593F and 618R primers and inserted into pG+host1^ts^ as described above. After allelic exchange, the *in situ* complementation of *SAK_1741* was confirmed by sequencing.

### β-galactosidase transcriptional fusion assays.

The promoter regions of *SAK_0348*, *SAK_0902*, and *SAK_1689* were cloned in a pTCV-*lacZ* (80) vector to construct transcriptional fusions between the E. coli *lacZ* reporter gene and upstream regions of the genes as described by Patron et al. (81). Briefly, promoter regions were amplified with the primers listed in Table S2. The DNA fragments and pTCV-*lacZ* vector were then purified and digested by EcoRI and BamHI before cloning. For β-galactosidase assays, bacteria were harvested in the mid-exponential phase (10 mL of culture for the A909WT and A909Δ*ccpA*::*ccpA* strains and 20 mL for the A909Δ*ccpA* mutant). Pelleted bacteria were suspended in 500 μL of Z-buffer for the WT and A909Δ*ccpA*::*ccpA* strains and 350 μL for A909Δ*ccpA* and lysed mechanically with glass beads in a FastPrep-24 instrument, and cell debris was eliminated by centrifugation (5 min; 8,000 × *g*) (82). Supernatants were used for assays. The absorbance at 595 nm (A_595_) and the absorbance after addition of *o*-nitrophenyl-β-d-galactopyranoside at 420 nm (A_420_) were measured as described by Patron et al. (81). Protein concentration (*C*_mg/mL_) was deduced from A_595_. β-Galactosidase activity was calculated in arbitrary units per milligram of protein using the following formula: (1,000 × *V*1 × A_420_)/(*V*2 × *t* × *C*_mg/mL_), with V1 being the volume of the sample that was added to the reaction mixture for β-galactosidase in milliliters, V2 being the volume of the sample that was added to the reaction mixture in milliliters, and *t* being the reaction time in minutes. The experiments were performed over four independent biological replicates.

### Acidic and oxidative stress.

For survival experiments under acidic stress, bacterial strains were grown in TH to the mid-exponential phase, and then 10 mL of the culture was pelleted and resuspended in 10 mL of TH adjusted to pH 4 with HCl. At time point zero (*t* = 0) and after 24 h, samples were taken from each culture, serially diluted with physiological saline (milli-Q water plus 0.85% NaCl), and plated on TH agar plates. Assays were performed over at least three independent biological replicates.

For survival experiments under oxidative stress, bacterial strains were grown in TH broth to the mid-exponential phase and 20 mM H_2_O_2_ was added. At time point zero (*t* = 0) and after 30 min, samples were taken from each culture, serially diluted with physiological saline (milli-Q water plus 0.85% NaCl), and plated on TH agar plates. Assays were performed over at least three independent biological replicates.

For transcriptional study of *SAK_1689* and *SAK_1741* under stress conditions, overnight cultures were subcultured (OD_600_, 0.05) in TH liquid medium. At the mid-exponential phase, bacterial pellets were resuspended in TH at pH 4, or 2.5 mM H_2_O_2_ (=MIC) was added in the culture. After 20 min at 37°C, cells were harvested by centrifugation at 7,000 × *g* for 10 min, and total RNAs were extracted for RT-qPCR with the primers listed in Table S2. The expression levels of the tested genes were normalized with the *recA* gene. Each assay was performed at least in technical duplicate and repeated with at least three biological independent RNA samples.

### Survival in macrophages.

Survival experiments in RAW 264.7 macrophages (ATCC TIB-71) were performed in 24-well plates. Cells were dispensed to 24-well plates at 4.10^5^ cells/well 24 h before the assay. Bacterial strains were grown in TH until they reached the mid-exponential phase. Cultures were washed in RAW medium (Dulbecco’s modified Eagle’s medium [DMEM; Gibco] supplemented with 10% Fetal Bovine Serum [Sigma-Aldrich], 2 mM l-glutamine [Gibco], and 1 mM Na pyruvate [Gibco]) and adjusted to the desired inoculum in RAW medium, and CFU counts were verified by plating serial dilutions onto TH agar plates. Macrophages were infected with S. agalactiae strains at a multiplicity of infection of ~15 in RAW medium at 37°C with 5% CO_2_ for 1 h to allow bacterial phagocytosis. Cells were washed twice in RAW medium and then incubated in RAW medium-gentamicin (500 μg/mL) (Gibco) for 2 h. Gentamicin (50 μg/mL) was added in the RAW medium until the *T*24 h. Time zero (*T*0) of the assay was determined as the time after incubation with antibiotic. Infected macrophages at *T*0 and *T*24 h were washed three times with phosphate-buffered saline (PBS) and then lysed with 1 mL ice-cold Milli-Q water for 30 min. CFU counts were determined by plating serial dilutions onto TH agar plates. Assays were performed over four technical replicates and repeated over at least two independent biological replicates.

### RNA extraction.

Bacterial pellets were resuspended in a buffer (glucose, 10%; Tris, 12.5 mM, pH 7.6; and EDTA, 10 mM) and lysed mechanically with glass beads in a FastPrep-24 instrument, and total RNAs were extracted using a phenol/TRIzol-based purification method (83). Total RNAs were then treated with DNase (Turbo DNA-free DNase; Ambion) according to the manufacturer’s instructions and then tested by PCR with *gyrB* primers to check for DNA contamination (Table S2).

### Reverse transcription and RT-qPCR.

The iScript cDNA synthesis kit (Bio-Rad) was used according to the manufacturer’s instructions to synthesize cDNA. Primers were designed with Primer3 software (http://bioinfo.ut.ee/primer3-0.4.0/) (84) in order to achieve a melting temperature (*T_m_*) of ≈60°C and to amplify ≈100-bp amplicons (Table S2). qPCRs were performed with 50 ng of cDNA, 0.33 μM gene-specific primers, and 1× LightCycler 480 SYBR green I master mix (Roche). PCR amplification, detection, and analysis were performed as described by Moulin et al. (76). The fold change in transcript level was calculated using the 2^–ΔΔ^*^CT^* method (85). For each oligonucleotide pair, the efficiency was checked with a genomic DNA (gDNA) standard of S. agalactiae A909WT and superior to 1.8.

### Strand-specific RNA-seq library preparation.

RNA integrity was verified with the Agilent Bioanalyzer 2100. mRNA enrichment using the MICROBExpress kit (Ambion) and preparation of strand-specific RNA-seq libraries using the Illumina primer ligation method were performed as previously described (86) on three independent biological experiments. Multiplexed libraries (6 samples per lane) were sequenced on the HiSeq 2000 platform (Illumina) with a read length of 50 nt.

### Data analysis.

The S. agalactiae A909 genome sequence (NC_007432.1) was used as a reference sequence to map trimmed reads using Bowtie (version 0.12.7) (87) as previously described (86). RNA-seq data were analyzed using Rsamtools (version 1.13.35), GenomicRanges (version 1.13.39), and GenomicFeatures (version 1.12.3) in R (version 3.0.1) as previously described (86). For differential expression analyses, normalization, and statistical analyses were performed using EdgeR software (version 3.2.4) (88); *P* values were adjusted for multiple testing using the false-discovery rate controlling procedure (89).

Annotation of unknown function coding genes of the S. agalactiae A909 genome sequence (NC_007432.1) was completed using the MicroScope platform (https://www.genoscope.cns.fr/agc/microscope) (90) and the Pfam (version 35.0) database (https://pfam.xfam.org/) (91). S. agalactiae A909 resulting proteins were mapped onto a set of metabolic pathways for S. agalactiae available at the Kyoto Encyclopedia of Genes and Genomes (KEGG Pathway) (https://www.kegg.jp/kegg/) (92) in order to group genes into functional classes.

### Validation of RNA-seq by RT-qPCR.

To confirm the data obtained in the RNA-seq, RT-qPCRs were performed on *rbsR* (*SAK_0171*), *ptsG* (*SAK_1920*), *glpK* (*SAK_0345*), *pyk* (*SAK_1037*), *ptsK* (*SAK_0862*), *ptsI* (*SAK_0946*), *budB* (*SAK_1279*), *pfkA* (*SAK_1036*), *ldhA* (*SAK_0821*), *adhP* (*SAK_0087*), *SAK_0674*, *covR* (*SAK_1639*), *pflB* (*SAK_1735*), and *ahpF* (*SAK_1854*) genes with the primers listed in Table S2, using RNA extracted under the same conditions as for the RNA-seq. The numbers of transcripts of each gene were normalized against transcript levels of two housekeeping genes (*gyrB* and *rpoB*). Three independent biological replicates with three technical replicates for each were performed.

### Bioinformatic analysis of *cre* sites.

For the prediction of CcpA binding sites on the S. agalactiae genome, the positional weight matrix (PWM)-based model of the streptococcal *cre* site proposed by RegPrecise (https://regprecise.lbl.gov/) was used. The motif WWGWAARCGNTTWCWW (W for A or T, R for A or G, N for any base) in the leader regions from position −500 to +500 bp relative to the translational start site, allowing no more than 2 mismatches, was searched in the entire genome of S. agalactiae A909 using the Virtual Footprint web server.

### Electrophoretic mobility shift assay.

Regions overlapping the defined *cre* site of *rbsR* (*SAK_0171*), *SAK_0257*, and *typA* (*SAK_0575*) genes were amplified by PCR using the primers listed in Table S2. The probes containing *SAK_1068* and *SAK_0473* coding regions were used as negative controls. The amplified DNA fragments were end-labeled with digoxigenin-11-ddUTP (81) according to the instructions of the manufacturer of the Dig Gel Shift kit, 2nd generation (Roche). DNA fragments and increasing amounts of purified CcpA-His_6_ (93) were incubated at 20°C and 250 rpm for 15 min. The resulting DNA-protein complexes were loaded onto a 7% nondenaturing polyacrylamide gel in Tris-glycine buffer (25 mM Tris-HCl, pH 8, 190 mM glycine, 1 mM EDTA). After a run of 1 h at 130 V, the gel was transferred by capillarity to a positively charged nylon membrane (Amersham). The latter was incubated with anti-digoxigenin-conjugated antibodies conjugated with alkaline phosphatase. Chemiluminescent DNA fragments were revealed with a PXi 4 CCD camera (Ozyme). Image acquisition was performed with Genesys software.

### ^1^H-NMR spectroscopy analysis.

Overnight cultures of strains A909WT, A909Δ*ccpA*, and A909Δ*ccpA*::*ccpA* were subcultured (OD_600_, 0.05) in CDM supplemented with 0.25% glucose. After 24 h, cultures were centrifuged at 7,000 × *g* for 10 min. The culture supernatants were collected and filtered. Then, 150 μL of culture supernatants was added with 50 μL of 0.2 M potassium phosphate buffer in 99% deuterium oxide (D_2_O) at pH 7.4. Samples were spiked with 10 μL of 3-trimethylsilylpropionic acid (3.2 mM in D_2_O) as an internal reference (Ref), and then samples were transferred to conventional 3-mm NMR tubes. ^1^H-NMR spectra were obtained with an AVANCE III HD 600 spectrometer equipped with a TCI cryoprobe (Bruker). Standard water-suppressed ^1^H-NMR spectra were acquired at 298 K using a “noesypr1d” pulse sequence with a relaxation delay of 20 s and 64 scans. Spectra were processed using Topspin software (Bruker). ^1^H-NMR spectra were automatically reduced to ASCII files using the AMIX software package (Analysis of Mixture, version 3.8, Bruker). Spectral intensities were scaled to the internal reference intensity, and then concentrations were calculated using the equation

$$\text{Concentration of compound}=\frac{\text{Intensity of compound}\cdot \text{Concentration of Ref}\cdot \text{nbr H Ref}}{\text{Intensity of Ref}\cdot \text{nbr H compound}}$$

Concentration Ref=10 μL⋅3.2mM/210μL=152μM

Nbr H Ref=9

Nbr H Ref, number of hydrogen of the internal reference; nbr H compound, number of hydrogen of the compound. The experiments were performed over three independent biological replicates with three technical replicates for each.

### Statistical analyses.

Analyses were performed using the one-sample *t* test, analysis of variance (ANOVA) test, unpaired *t* test, or nonparametric Wilcoxon test. A probability value of less than 0.05 was considered statistically significant.

### Data availability.

RNA-seq data are available in the ArrayExpress database (http://www.ebi.ac.uk/arrayexpress) under accession number E-MTAB-11639.
