# Supplementary material for: The Role of Regulator Catabolite Control Protein A (CcpA) in Streptococcus agalactiae Physiology and Stress Response
Source: Microbiol Spectr. 2022 Oct 20;10(6):e02080-22. doi: 10.1128/spectrum.02080-22 (PMC9784791; doi:10.1128/spectrum.02080-22)
Supplement: Supplemental file 1 — Supplemental material. Download spectrum.02080-22-s0001.pdf, PDF file, 1.4 MB [file spectrum.02080-22-s0001.pdf]

MATERIALS AND METHODS

RT-qPCR according the sugar contents in the medium

To measure transcripts expression of *ccpA* (*SAK_0833*), *rbsR* (*SAK_0171*), *ptsG* (*SAK_1920*) and *pyk* (*SAK_1037)* genes according to the different sugar contents in the medium, A909WT strain was subcultured ($OD_{Abs=600nm}$ = 0.001) in TH or in CDM supplemented with different sugars (0.25% glucose, 1% glucose, 2% glucose, 1% fructose, 1% saccharose, 1% ribose, 1% galactose or four sugars together: 0.5% ribose + 0.5% arabinose + 0.5% fructose + 0.5% saccharose (4SS)) until mid-exponential phase. Then total RNA was extracted as explained in the "Materials and Methods" section and RT-qPCR were performed with the primers listed in the Table S2. The amount of transcripts of three independent biological replicates with two technical replicates of each gene was normalized against transcript levels of the *recA* housekeeping gene.

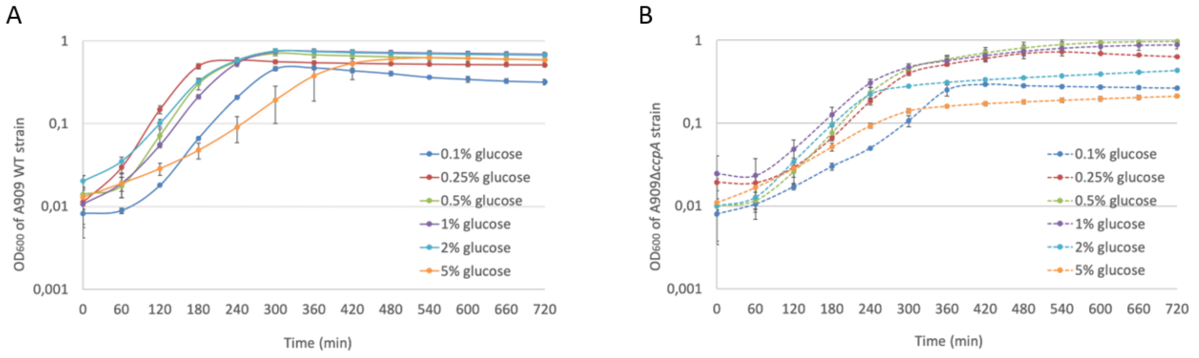

Figure S1. **Growth curves of *S. agalactiae* with increased glucose concentrations**. (A) Growth of A909WT and (B) A909Δ*ccpA* strains in CDM medium supplemented with 0.1, 0.25%, 0.5%, 1%, 2% or 5% glucose in 96-well microplates. The curves are means ± SD over at least two independent biological replicates with three technical replicates for each.

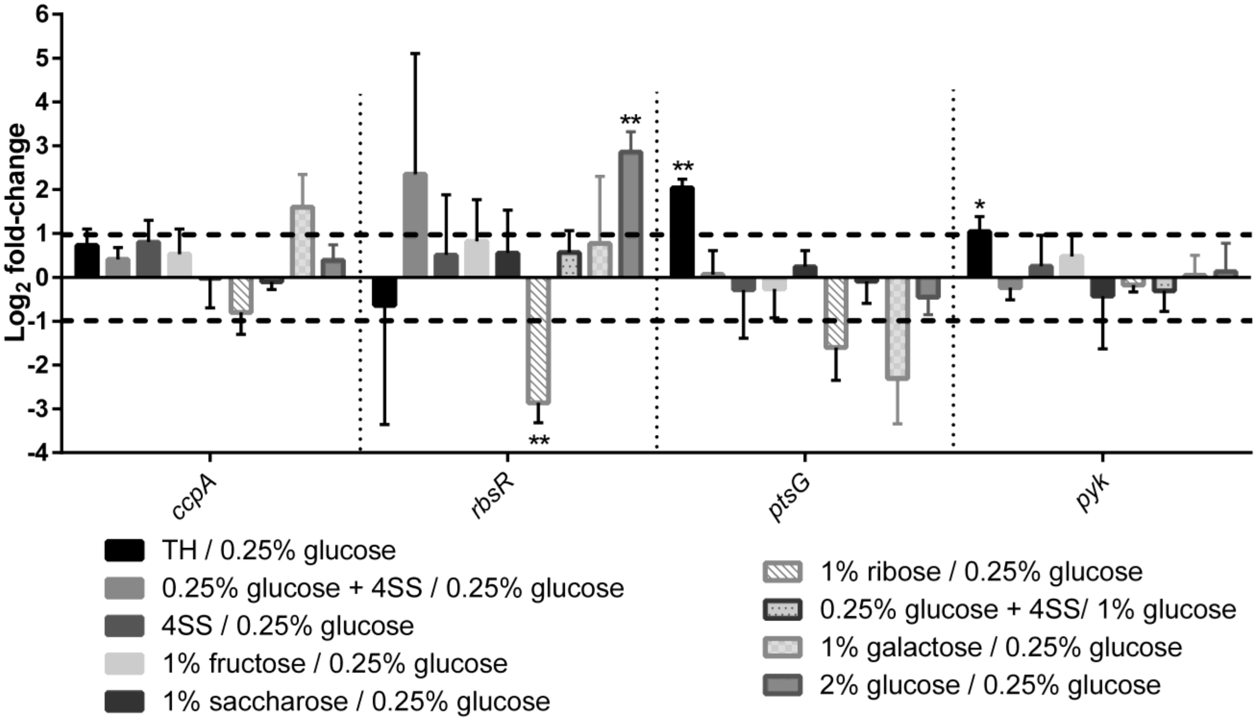

Figure S2. **Gene expression according to the different sugar contents in the medium.** The transcript levels of *ccpA* (*SAK_0833*), *rbsR* (*SAK_0171*), *ptsG* (*SAK_1920*), *pyk* (*SAK_1037)* genes of the A909WT strain were detected by RT-qPCR after growth in media with different sugar contents in order to highlight the “glucose effect”. The strain was grown in TH medium or in CDM supplemented with different sugars (0.25% glucose, 1% glucose, 2% glucose, 1% fructose, 1% saccharose, 1% ribose, 1% galactose or four sugars together: 0.5% ribose + 0.5% arabinose + 0.5% fructose + 0.5% saccharose (4SS)). The amount of transcripts of each gene was normalized against transcript levels of the *recA* housekeeping gene. Gene expression is presented as the Log_2 fold-change in gene expression between two different conditions. The data are means ± SD of three independent biological replicates with two technical replicates for each. The dotted lines represent a Log_2 fold-change of -1 and 1. The asterisks indicate *P* values obtained using one-sample *t*-test. *, *P*<0.05 and **, *P*<0.01.

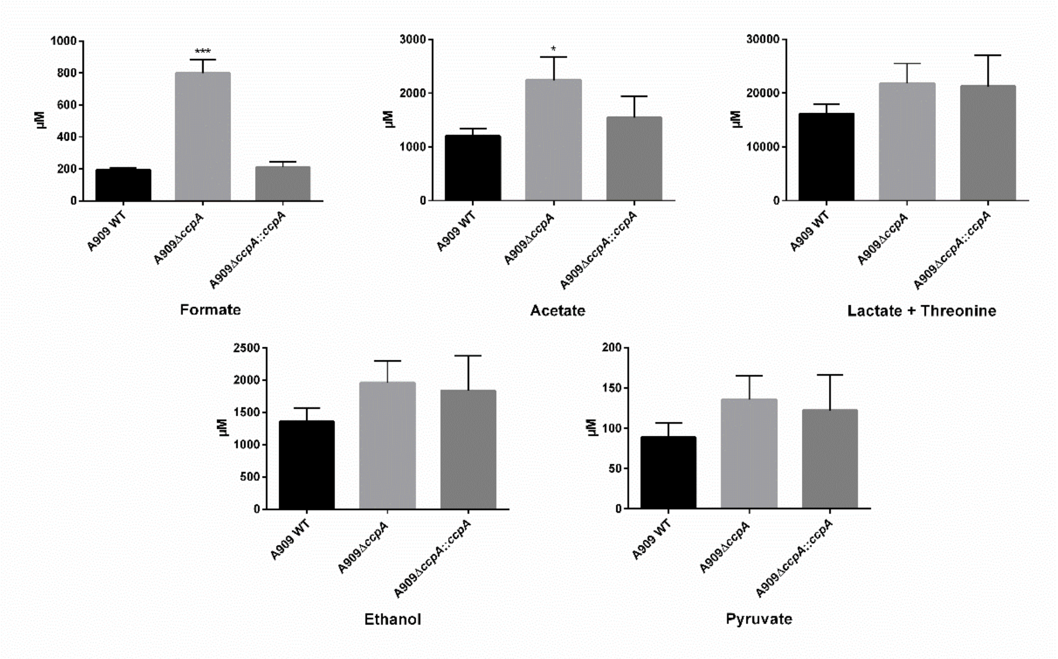

Figure S3. **Fermentation end products of the strains A909WT, A909Δ*ccpA* and A909Δ*ccpA*::*ccpA* in CDM with glucose.** NMR spectroscopy analysis of the metabolic end products from strains A909WT, A909Δ*ccpA* and A909Δ*ccpA*::*ccpA* after 24 h of growth in CDM supplemented with 0.25% glucose was performed. The data are means + SD over three independent biological experiments performed with three technical replicates for each. The asterisks indicate *P* values obtained using ANOVA and then unpaired *t*-test to compare the value of strains A909Δ*ccpA* and A909Δ*ccpA*::*ccpA* to the strain A909WT. *, *P*<0.05 and ***, *P*<0.001.

A

```
lmo1580     MLQQYERVLVAVDGSKEAERAFQKAIQVANRNDAALGLVHVIDTRAFSSVANYDTSMADK    60
SAK_1689    MSQKYERILVAIDGSTESELAFEKAVNVALRNDSELILTHVIDTRALQSFATFDTYIYEK    60
SAK_1741    MTQKYERILIAIDGSYESELAVEKGINVALRNDAELLLTHVIDAHAYQSEGVFSDYVFDR    60
            * *:***:*:*:*** *:* *.:*.::** ***: * *.****::* .* . :.  : ::

lmo1580     ATEYADELLSGYKEDALKAGVTKVESYIEYGSPKTAITKEAAKAFQADLIMCGATGLNAV    120
SAK_1689    LEKEAKDVLEEYEKQAREKGADKVRQVIEFGNPKTLLAHDIPEKEKVDLIMVGATGLNTF    120
SAK_1741    QEQESADVLAYFEKLAHSKGLTKIKKITEIGNPKTLLAKDIPIREKADLIMVGATGLNTF    120
              : : ::*  ::: * . *  *:..  * *.*** ::::     :.**** ******:.

lmo1580     ERLLIGSVSEYIIRHSPCDVLVVRNEVPDYKDEK*    154
SAK_1689    ERFLIGSSSEYILRHAKVDLLIVRDPNKTM*----    150
SAK_1741    ERLLIGSTSEYILRHSKVDMLVVRDSKKTL*----    150
            **:**** ****:**:  *:*:**:
```

(*): identity, (:): strongly similar, (.): weakly similar

B

	Lmo1580	Lmo0515	Lmo2673	SAK_1689	SAK_1741
Lmo1580		**45%** 69%	**45%** 68%	**52%** 70%	**49%** 68%
Lmo0515	**45%** 69%		**42%** 59%	**44%** 63%	**40%** 61%
Lmo2673	**45%** 68%	**42%** 59%		**37%** 57%	**30%** 51%
SAK_1689	**52%** 70%	**44%** 63%	**37%** 57%		**67%** 81%
SAK_1741	**49%** 68%	**40%** 61%	**30%** 51%	**67%** 81%	

Figure S4. **Comparaison of the proteins Lmo1580, Lmo0515, Lmo2673 of *Listeria mocytogenes* EGD-e and SAK_1689 and SAK_1741 of *S. agalactiae* A909.** (A) Protein alignment of SAK_1689, SAK_1741 and Lmo1580 was performed with Clustal Omega websoftware (https://www.ebi.ac.uk/Tools/msa/clustalo/) (B) Determination of percent identity (in bold) and similarity of Lmo1580, Lmo0515, Lmo2673, SAK_1689 and SAK_1741 proteins. Percentage of identity and similarity were performed with BLASTP® websoftware (https://blast.ncbi.nlm.nih.gov/Blast.cgi?PAGE=Proteins).

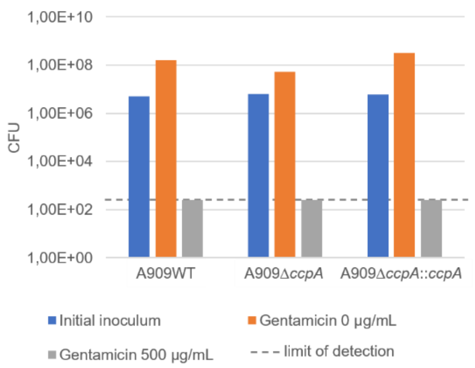

B

	T0 plate		T24 plate	
	Before infection	After infection and Gentamicin (500 µg/mL) during 2h	Before infection	Gentamicin (500 µg/mL) during 2h and gentamicin (50 µg/mL) during 24h
RAW 264.7 Pictures (InCellis instrument)	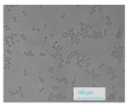	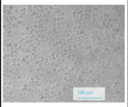	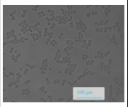	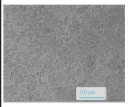
Confluency (%)	33	60	33	91
Cell count (n/well)	$3.6x10^5$	$6.5x10^5$	$3.6x10^5$	$9.9x10^5$

Figure S5. **Experimental controls for bacterial survival in macrophages.** (A) counts of *S. agalactiae* tested strains (CFU) after 1h of growth in RAW medium + 2h in absence (orange) or in presence (grey) of gentamicin 500µg/mL in 96-well plate without macrophage, with a limit of detection at 260 CFU. (B) Analysis of macrophages RAW 264.7 at key times of survival experiments (before infection, after antibiotic treatment and after 24h of infection) by controlling their morphology (Pictures with InCellis Instrument), confluency and concentration (cells/well).

Table S1. Bacterial strains and plasmids used in this study		
***E. coli* strains**		
XL1-blue	*endA1 gyrA96* (Nal^R) *thi-1 recA1 relA1 lac glnV44 hsdR17*($r^-_k m^+_k$) *F′* [::Tn10 (Tet^R) *proAB*$^+$ $lacI^q$ ZΔM15]	Stratagene
BL21 codon + (DE3)-RIL	F- *dcm ompT hsdS* (r_B- m_B-) *gal* [*malB*$^+$]$_{K-12}$(λ^S)	Stratagene
***S. agalactiae* strains**		
A909	Isolated from a septic human neonate in 1934	(1)
A909Δ*ccpA*	Isogenic *ccpA* (*SAK_0833*) deletion mutant of A909	This study
A909Δ*ccpA::ccpA*	*ccpA* (*SAK_0833*) *in situ* chromosomal complementation of A909Δ*ccpA*	This study
A909Δ*SAK_1689*	Isogenic *SAK_1689* deletion mutant of A909	This study
A909*SAK_1689*::*SAK_1689*	*SAK_1689 in situ* chromosomal complementation of A909Δ*SAK_1689*	This study
A909-SAK_1741^{Y5STOP}	Isogenic substitution mutant of A909 coding a STOP codon at position 5 of *SAK_1741* in place of a tyrosine	This study
A909-SAK_1741^{Y5STOP}::*SAK_1741*	*SAK_1741 in situ* chromosomal complementation of A909-SAK_1741^{Y5STOP}	This study

A909Δ*SAK_1689*-SAK_1741^{Y5STOP}	Isogenic double *SAK_1689* deletion and *SAK_1741* substitution mutant of A909	This study
A909Δ*ccpA*-Δ*SAK_1689*	Isogenic double *ccpA* deletion and *SAK_1689* deletion mutant of A909	This study
**Plasmids**		
pET28a	Vector for expression of His-Tagged proteins, Kan^R	EMD Biosciences
pET28a:: *ccpA*	pET28a containing the *ccpA* gene of *S. agalactiae* A909 in the BamHI/EcoRI restriction sites	(2)
pG+host1^{ts}	Replication-thermosensitive shuttle plasmid, Ery^R	(3)
pTCV-*lacZ*	Promoter probe plasmid carrying a *lacZ* gene devoided of a promoter and carrying the *ermB* gene (Ery^R)	(4)
pTCV-P_{SAK_0348}*::lacZ*	pTCV-*lacZ* containing the promoting region of *S. agalactiae* A909 *SAK_0348* gene in the EcoRI/BamHI restriction sites upstream of the *lacZ* gene	This study
pTCV-P_{cshA}*::lacZ*	pTCV-*lacZ* containing the promoting region of *S. agalactiae* A909 *cshA* (*SAK_0902)* gene in the EcoRI/BamHI restriction sites upstream of the *lacZ* gene	This study

pTCV-P$_{SAK_1689}$::*lacZ*	pTCV-*lacZ* containing the promoting region of *S. agalactiae* A909 *SAK_1689* gene in the EcoRI/BamHI restriction sites site upstream of the *lacZ* gene	This study

Table S2. Primers used in this study			
**Name**	**Sequence[a]**	**Location[b]**	**Gene**
**Primers used for deletion and for complementation (*)**			
65F (*)	GAACAGGAATTCCAGCAAATTCTCCAGCGACT	nt -657	*ccpA (SAK_0833)*
66R	CATACTCTAACACTACGTATCATCTGTATTCATATTG	nt -4	*ccpA (SAK_0833)*
67F	AATACAGATGATACGTAGTGTTAGAGTATGTTATTTG	nt +1003	*ccpA (SAK_0833)*
68R (*)	TTCATCGGATCCCAAATCATCGTCTGCCCAACC	nt +1680	*ccpA (SAK_0833)*
602F (*)	TGAAGGATCCCCTTTCGAATCAGAGTAACC	nt -484	*SAK_1689*
601R	TTTACATTGTTTGTGACATAAGCCTGTCC	nt -10	*SAK_1689*
600F	TATGTCACAAACAATGTAAATTTAAATACGCTC	nt +445	*SAK_1689*
599R (*)	ATGAGAATTCGGAAGCTGGTTTCAGGTCA	nt +920	*SAK_1689*
593F (*)	GATCCGAATTCCCTATGCTTCCGTGTTAGGT	nt -534	*SAK_1741*
616R	GAATACGTTATTATTTTTGTGTCATAGCTTAATTC	nt +25	*SAK_1741*
617F	GAATTAAGCTATGACACAAAAATAATAACGTATTC	nt -10	*SAK_1741*
618R (*)	TAGTGGATCCGGTTTTCTTACTATCACGAACG	nt +447	*SAK_1741*
**Primers used for verification of the mutants by sequencing**			
1F	GCCAGTCAAAACCATAGTGTCG	nt -791	*ccpA (SAK_0833)*
4R	CAATGTCATTGTACATGAGATAATC	nt +1749	*ccpA (SAK_0833)*
565F	CCAGCAAGGCTAACACAAG	nt -682	*SAK_1689*
566R	GATGATGGTATCTCTAAGGC	nt +1077	*SAK_1689*
597F	AATTGCGATTGTAGTGTTGGG	nt -699	*SAK_1741*
598R	ACCAGCTTGAACAAGTTTACC	nt +1170	*SAK_1741*
**Primers used for transcriptional fusion and for electrophoretic mobility shift assays**			
585F	CTATGGAATTCGAGATTTTTGATGCTGAGACC	nt -87	*SAK_0348*
586R	CTTGGATCCCTACATACCAGCAAAAGAAGCTCC	nt +40	*SAK_0348*
591F	TTGCGGAATTCCTATGAACCCTCCTTCTTTAT	nt -225	*cshA (SAK_0902)*
592R	TCTGGATCCTTACAAATGTTTCTCTTTTCTAAAAG	nt -20	*cshA (SAK_0902)*
583F	CCTAGAATTCCTTTTACTGCGGTTTATCAAGC	nt -216	*SAK_1689*

584R	CATAGGATCCTTACATAAGCCTGTCCTCCTCTAA	nt -18	*SAK_1689*
324F	GGTTTTTCTCCTTTGATC	nt -181	*rbsR (SAK_0171)*
323R	AAAAACTCCTTTCTTTTATTGT	nt -22	*rbsR (SAK_0171)*
448F	GAGGGTGACTGATCTACTCA	nt -231	*SAK_0257*
449R	CCCCAATTGCCTCTAACAAG	nt +30	*SAK_0257*
482F	GCTGCTGTTGTTGCTGTTAAA	nt -192	*SAK_0473*
483R	CGACTTCCCGCATCTATTA	nt +98	*SAK_0473*
436F	ATTACTGGACGGGTAGAGTT	nt -257	*typA (SAK_0575)*
437R	TCAACGTGGGCAATGATCG	nt +35	*typA (SAK_0575)*
490F	AGGGACTCATTTTAGACTTATC	nt +476	*SAK_1068*
491R	AAGTCATTTCTGCCGAACTAG	nt +704	*SAK_1068*
**Primers used for RT-qPCR**			
200F	GCGGACTATGCGGTTAAGGT	nt +376	*adhP (SAK _0087)*
201R	ACCACCTGCACCATACACTG	nt +500	*adhP (SAK _0087)*
93F	GACCACCATCCAGACATTGA	nt +676	*rbsR (SAK _0171)*
94R	TTCGCTTCCCTCGATTAAGA	nt +747	*rbsR (SAK _0171)*
19F	TATTGAAAAAGACATTCATAAAAATCCA	nt +660	*rpoB (SAK _0223)*
20R	CAAAGAAACGTGCAACTAAAAGACTAC	nt +770	*rpoB (SAK _0223)*
147F	CAAGTGACGATGAGGTTTATGTG	nt +1016	*glpK (SAK _0345)*
148R	CCCACGAGTTAATCCAAATACTG	nt + 1094	*glpK (SAK _0345)*
208F	GCCCCAACAACTCCTCTTGA	nt +262	*SAK _0674*
209R	ATGTTTCTGAGCTGCTTGGC	nt +347	*SAK _0674*
21F	GCTCACATCAGAACTTTACTTTTAACTC	nt + 1519	*gyrB (SAK _0708)*
22R	TTTAATCTCACTTCCTACTTTGACACC	nt +1615	*gyrB (SAK _0708)*
194F	GTGCCAAGTTACTCACCGGA	nt +289	*ldhA (SAK _0821)*
195R	TCCTCGAATTGGCAAGGTCC	nt +401	*ldhA (SAK _0821)*
9F	AGCGGCAGGTTTGTTAAATG	nt +424	*ccpA (SAK_0833)*
10R	ATTGGCGAGTCATTGCTTGT	nt +820	*ccpA (SAK_0833)*
113F	CGGTTAAAGATTCATCGCAAG	nt +656	*ptsK (SAK _0862)*

114R	GCCATTCCCGAGTCTATCAA	nt +722	*ptsK (SAK _0862)*
81F	CAGTTCTTGAGGGTATGAATGGTAA	nt +962	*ptsI (SAK _0946)*
82R	TCTCTTTTGGAAGATCAAAGTAAGG	nt +1034	*ptsI (SAK _0946)*
202F	TTGGTCAGGTATCGCTGCAG	nt +534	*pfkA (SAK _1036)*
203R	GCTATCGTCTCCTGCTGCTT	nt +704	*pfkA (SAK _1036)*
196F	ATTCCGTCCAGATGCAGACA	nt +1254	*pyk (SAK _1037)*
197R	TGCTACACGTTCTGCAACCT	nt +1379	*pyk (SAK _1037)*
206F	TCGTGTAGGCCTCTTCCGTA	nt +765	*budB (SAK _1279)*
207R	ATCAGTTCTCGCTCGGGTTG	nt +938	*budB (SAK _1279)*
224F	AAATACGATGTGGCAGCAGAGA	nt +556	*covR (SAK _1639)*
225R	CCATTCCGCGAACAGTTTGAAT	nt +646	*covR (SAK _1639)*
567F	TCTTAATACCTTTGAACGTTTCCTTATCG	nt +348	*SAK_1689*
568R	TTACATTGTTTTATTAGGGTCACGTACAAT	nt +424	*SAK_1689*
212F	ACCTGATCCAGCTGTTCACG	nt +387	*pflB (SAK _1735)*
213R	GTAACAGTGTGTGCATGGCG	nt +481	*pflB (SAK _1735)*
569F	ATTACTGAAATCGGAAATCCTAAGACTCTT	nt +259	*SAK_1741*
570R	GAATGACGTAAAATGTATTCTGAAGTTGAG	nt +377	*SAK_1741*
222F	AAGGCCCTAAATTGATGGCTCA	nt +782	*ahpF (SAK _1854)*
223R	CCAGGAACGTTGATATTTCGCC	nt +953	*ahpF (SAK _1854)*
91F	TAGCAGCAGCCCAAGTTACA	nt +1697	*ptsG (SAK _1920)*
92R	CAGTTGGTTCCACAGCAAAA	nt +1824	*ptsG (SAK _1920)*
319F	ATTCAGGCGCAGTTGATTTAGTT	nt +323	*recA (SAK _2032)*
320R	TCAATCTCAGCACGAGGAACA	nt +369	*recA (SAK _2032)*

a. Tails containing a restriction site are underlined

b. Nucleotide (nt) position with respect to the first coding nucleotide of the gene of interest

**Table S3. Genes regulated by CcpA in mid-exponential phase in a chemically defined medium supplemented with 0.25 % glucose**						
**Gene number**	**Gene name**	**Putative function of encoded protein**	**Log2 Fold Change (Δ*ccpA*/WT)**	***P*-value**	**Position of the *cre* site from START codon**	***cre* sequence** *: Virtual Footprint only **: RegPrecise only
**Cell enveloppe**						
SAK_0252	-	peptide/opine/nickel uptake ABC transporter substrate-binding protein	1,68	6,25E-04		
SAK_0253	-	peptide/opine/nickel uptake ABC transporter permease	2,51	8,04E-05		
SAK_0254	-	peptide/opine/nickel uptake ABC transporter permease	2,55	1,80E-05		
SAK_0255	-	peptide/opine/nickel uptake ABC transporter ATP-binding protein	2,05	2,69E-04	Pos=+62	TTGAAATGGTTTTAAA**
SAK_0256	-	peptide/opine/nickel uptake ABC transporter ATP-binding protein	2,07	2,93E-05		
SAK_0299	-	major facilitator family transporter	-1,87	5,72E-04	Pos=+203	AAGAAAAATTTTACTA*
SAK_0362	-	polar amino acid ABC transporter amino acid-binding protein	-1,41	3,03E-02		
SAK_0685	*adcA*	zinc ABC transporter, zinc-binding protein AdcA	-1,72	2,65E-03	Pos=-95	ATGCAAGCGTTTTTAT*
SAK_0698	-	ABC transporter, permease protein	-2,81	4,62E-02	Pos=+127	TATAAAGCCTTTTCTA*
SAK_0771	-	cell wall surface anchor family protein, truncation	3,84	1,54E-05		

SAK_0781	-	pseudo-sortase	1,39	2,27E-02		
SAK_0787	-	ABC transporter, ATP-binding protein	4,08	2,13E-16	Pos=-115	AAGAAAACGTTTGCAT
SAK_0788	-	hypothetical protein	4,49	4,52E-16	Pos=-230	AAGAAAGAGTTTTCAT*
SAK_0789	-	hypothetical protein	4,10	1,07E-12		
SAK_0840	-	peptidase propeptide/YPEB domain-containing protein	-1,46	6,76E-03		
SAK_0890	-	penicillin-binding protein 2b	-1,15	3,40E-02		
SAK_0900	-	amino acid ABC transporter, permease protein, putative	1,93	5,72E-03		
SAK_0901	-	ABC transporter, substrate-binding protein	1,94	3,37E-03		
SAK_0958	-	hypothetical protein	2,97	2,88E-02		
SAK_1027	-	cation transporter HAD ATPase	2,07	2,81E-04	Pos=-16	TCTAAAACGTTTTCAT*
SAK_1125	-	Type VII secretion accessory protein	-2,33	2,20E-02	Pos=-341	TACAAAACGTTTAGAA*
SAK_1128	-	Type VII effector, ESAT6 like protein	1,31	3,64E-02		
SAK_1394	-	RND family efflux transporter MFP subunit	-1,90	3,49E-04	Pos=-229	ATGAAAAATTTTTCTA*
					Pos=-116	TAGAAAGAACTTACAA*
SAK_1504	-	amidase family protein	1,36	1,27E-02	Pos=-20	ATGTAAACGTATACTT*
SAK_1517	-	ABC transporter, ATP binding protein	-1,23	2,26E-02	Pos=-15	TATAAAAGGTTTACTT*
SAK_1626	-	amidase	-2,63	7,47E-03		
SAK_1897	-	streptococcal histidine triad family protein	-4,36	3,37E-02		
**Cell division**						
SAK_0013	*tilS*	tRNA(Ile)-lysidine synthetase	-1,49	2,89E-02		
SAK_0408	*yfiA*	ribosomal subunit interface protein	3,46	1,70E-07	Pos=-36	TAGAAAGCGTTAACAA
					Pos=-60	ATGAAAGCGGTTATAA**

SAK_0575	*typA*	GTP-binding protein TypA	-1,68	3,89E-04	Pos=-96	ATGAAAACATTTACAC
SAK_0576	-	hypothetical protein	-1,49	9,81E-03		
SAK_1290	-	tRNA (adenine(22)-N(1))-methyltransferase	-2,01	4,14E-03	Pos=+29	ATCAAAACGATTACAA*
SAK_1589	-	DNA replication intiation control protein YabA	-1,41	1,30E-02		
SAK_1707	*efp*	elongation factor P	-1,27	3,51E-02		
SAK_1773	-	DNA polymerase III subunit epsilon	-3,77	1,17E-03		
**Amino acids metabolism**						
SAK_0088	*thrC*	threonine synthase	2,83	1,82E-08		
SAK_0176	*argG*	argininosuccinate synthase	2,92	9,19E-03		
SAK_0768	*aphA*	acid phosphatase/phosphotransferase	2,66	2,70E-06	Pos=-80	ATGATATCGCTTTCAT
SAK_1132	*carB*	carbamoyl phosphate synthase large subunit	-4,03	2,32E-03		
SAK_1133	*carA*	carbamoyl phosphate synthase small subunit	-5,31	3,08E-05		
SAK_1134	*pyrB*	aspartate carbamoyltransferase catalytic subunit	-5,12	9,00E-04		
SAK_1135	*pyrC*	dihydroorotase	-2,09	1,49E-02		
SAK_1136	*pyrE*	orotate phosphoribosyltransferase	-3,82	7,26E-04		
SAK_1137	*pyrF*	orotidine 5'-phosphate decarboxylase	-4,51	4,30E-04		
SAK_1211	-	aldehyde dehydrogenase family protein	4,63	2,30E-08	Pos=-53	TTGTAATCGATTTCAT
SAK_1431	*pflA*	pyruvate formate-lyase-activating enzyme	1,79	7,40E-03	Pos=-25	ATAAAAACGGTTACAA
					Pos=-47	ATGAAAATGATATAAT**
SAK_1502	*pepS*	aminopeptidase PepS	1,83	2,96E-03		
SAK_1685	-	3-hydroxybutyryl-CoA dehydrogenase	2,05	1,08E-03		
SAK_1691	-	asparaginase family protein	1,59	1,82E-02		

SAK_2060	-	peptidase propeptide/YPEB domain-containing protein	-2,03	4,60E-02		
SAK_2064	*arcC*	carbamate kinase	2,81	1,82E-03		
SAK_2065	*argF*	ornithine carbamoyltransferase	2,66	1,26E-03	Pos=-36	TAGATAGCGCTTTCAA*
SAK_2121	*arcA*	arginine deiminase	3,67	8,14E-03	Pos=-177	TAGAAATCGCTTTCAT
					Pos=-324	TACAAATCGCTTTCTA*
SAK_2122	-	acetyltransferase	4,68	9,58E-06		
SAK_2123	*argF*	ornithine carbamoyltransferase	6,83	7,56E-12		
SAK_2124	-	arginine/ornithine antiporter	5,53	4,06E-08		
SAK_2125	*arcC*	carbamate kinase	6,04	3,65E-07		
**Carbon and energy metabolism**						
SAK_0086	-	bifunctional acetaldehyde-CoA/alcohol dehydrogenase	3,76	5,18E-03	Pos=-146	ATTAAAGCGCTTTCAA
SAK_0087	-	alcohol dehydrogenase	7,61	9,55E-27	Pos=-49	ATGTAAACGATTACAA
SAK_0166	*rbsB*	ribose ABC transporter, ribose-binding protein	3,89	1,97E-07		
SAK_0167	*rbsC*	ribose ABC transporter, permease protein	3,49	5,45E-06		
SAK_0168	*rbsA*	ribose ABC transporter, ATP-binding protein	3,06	8,71E-05		
SAK_0169	*rbsD*	D-ribose pyranase	3,63	2,40E-02		
SAK_0170	*rbsK*	ribokinase	4,79	5,71E-10		
SAK_0171	*rbsR*	ribose operon repressor	6,05	2,48E-12	Pos=-60	TAGTAAGCGATTACAA
SAK_0234	*ackA*	acetate kinase	2,21	1,05E-04	Pos=-468	ATTACATCGCTTTCTT**
SAK_0257	-	PTS system, trehalose-specific IIBCA component	6,64	4,44E-22	Pos=-93	ATGTAATCGTTTACAA
SAK_0258	-	alpha amylase family protein	4,82	4,22E-14		
SAK_0261	*ulaA*	ascorbate-specific PTS system enzyme IIC	2,03	5,58E-03		

SAK_0263	-	transketolase, C-terminal subunit, putative	1,25	2,68E-02		
SAK_0345	*glpK*	glycerol kinase	4,04	1,58E-14	Pos=-31	AAGAAAACGCTTACTT
SAK_0346	*glpO*	glycerol-3-phosphate oxidase	4,94	4,32E-16		
SAK_0347	*glpF*	glycerol uptake facilitator protein	5,53	8,93E-15		
SAK_0350	*tkt*	transketolase	1,37	3,35E-02		
SAK_0433	-	PTS system mannose/fructose/sorbose family IIDsubunit	2,62	7,26E-04		
SAK_0434	-	PTS system lactose/cellobiose family IIC subunit	2,66	1,47E-03		
SAK_0435	-	PTS system mannose/fructose/sorbose family IIA subunit	1,96	1,64E-03	Pos=-130	CTGAAAACGTTTTAAA
					Pos=-248	TAGAAATCGTTTTTTA*
SAK_0661	-	Cof-like hydrolase (sugar phosphatase)	-1,55	1,57E-02		
SAK_0666	*fbp*	fructose-1,6-bisphosphatase	1,43	1,88E-02	Pos=-62	TAGAATGCGCTTTCAT
SAK_0674	-	acetoin reductase	3,77	4,37E-09	Pos=-131	TTGACACCGTTTTCAT
SAK_0826	*eda*	2-dehydro-3-deoxyphosphogluconate aldolase/4-hydroxy-2-oxoglutarate aldolase	1,82	2,99E-02	Pos=-29	AAGATAACGGTTACAT
SAK_0827	*uxaC*	glucuronate isomerase	2,37	3,90E-05		
SAK_0828	*uxuA*	mannonate dehydratase	3,19	2,92E-08		
SAK_0829	-	D-mannonate oxidoreductase	2,90	3,57E-08		
SAK_0831	-	glycosy hydrolase family protein	1,49	2,29E-03		
SAK_0862	*hprK*	HPr kinase/phosphorylase	1,79	1,97E-04	Pos=-68	TACTAAACACTTTCAT*
SAK_0915	*bglF*	PTS system, beta-glucoside-specific IIABC component	2,10	1,70E-04		
SAK_0919	-	GntP family permease	1,49	3,90E-02		
SAK_0975	*pulA*	pullulanase, type I	1,60	2,84E-02		
SAK_0976	*glgB*	glycogen branching enzyme	4,03	6,36E-10		
SAK_0977	*glgC*	glucose-1-phosphate adenylyltransferase	4,25	5,91E-09		

SAK_0978	*glgD*	glucose-1-phosphate adenylyltransferase, GlgD subunit	4,78	2,09E-10		
SAK_0979	*glgA*	glycogen synthase	4,70	2,36E-11		
SAK_1001	-	acetoin dehydrogenase, TPP-dependent, E1 component, alpha subunit, putative	1,59	1,58E-02	Pos=-81	AAGAAAGCGGTTCAAA
					Pos=-295	ATGAAAACAATATCAA*
SAK_1002	-	acetoin dehydrogenase, TPP-dependent, E1 component, beta subunit, putative	1,63	7,66E-03		
SAK_1003	-	branched-chain alpha-keto acid dehydrogenase subunit E2	1,34	2,11E-02		
SAK_1004	-	acetoin dehydrogenase, TPP-dependent, E3 component, dihydrolipoamide dehydrogenase, putative	1,48	2,98E-02		
SAK_1036	*pfkA*	6-phosphofructokinase	-2,23	7,07E-05		
SAK_1037	*pyk*	pyruvate kinase	-2,36	1,54E-05		
SAK_1155	-	phosphoglucomutase/phosphomannomutase family protein	2,11	5,11E-04	Pos=-35	ATGAAAGCGTTTATAA
SAK_1177	*pta*	phosphotransacetylase	1,54	5,80E-03	Pos=-39	TTGACAGCGTTATCAT*
SAK_1377	-	PTS system, fructose-specific IIABC component	1,87	5,58E-03		
SAK_1457	-	glycosyl transferase, group 2 family protein	-1,23	2,71E-02		
SAK_1475	-	carbohydrate ABC transporter periplasmic-binding protein	1,86	2,89E-02	Pos=-58	TTGCAAACGGTTGCAT
SAK_1476	-	cyclodextrin ABC transporter, permease protein	2,96	1,84E-05		
SAK_1477	-	cyclodextrin ABC transporter, permease protein	3,36	1,02E-06		
SAK_1651	-	alcohol dehydrogenase, zinc-containing	7,53	2,07E-22	Pos=-66	TTGTAACCGCTTTCTT
SAK_1682	*ppdK*	pyruvate phosphate dikinase	1,44	1,26E-02		
SAK_1683	*ppsR*	bifunctional ADP-dependent kinase-Pi-dependent pyrophosphorylase / positive regulator of gluconeogenesis	-1,55	2,67E-02		
SAK_1693	-	aldo/keto reductase family oxidoreductase	1,67	3,05E-03	Pos=-82	TAGTAAGCCTTTTCAT
SAK_1702	-	PTS system, sucrose-specific IIABC component	3,12	3,58E-07	Pos=-37	AATGAAACGCTTTCAA
					Pos=-146	TAGTAAACGTTTTCTC

SAK_1735	*pflB*	formate acetyltransferase 1	3,58	9,19E-06	Pos=-60	AAGTAAACGTTTTCGC
SAK_1756	*tkt*	transketolase	2,07	1,32E-03		
SAK_1757	-	putative translaldolase	1,91	1,51E-02		
SAK_1759	-	PTS system, fructose-specific, IIC component	1,63	2,27E-02		
SAK_1823	-	carbohydrate kinase	5,60	1,34E-08		
SAK_1824	-	hypothetical protein	5,67	8,79E-23		
SAK_1825	-	PTS system, IIC component, putative	6,29	1,28E-21	Pos=-20	ATGTAAGCGCTTAATA*
SAK_1893	-	PTS system, IIC component, putative	3,03	2,11E-08		
SAK_1894	-	PTS system, IIB component, putative	2,86	4,17E-03		
SAK_1895	-	PTS system, IIA component, putative	2,85	5,69E-03	Pos=-20	ATGTAACCGCTTAACT*
SAK_1908	-	PTS system mannose/fructose/sorbose family IIDsubunit	5,43	5,33E-17		
SAK_1909	-	PTS system mannose/fructose/sorbose family IIC subunit	5,27	1,12E-10		
SAK_1910	-	PTS system mannose/fructose/sorbose family IIB subunit	4,69	2,48E-12	Pos=-85	TTGTAACCGTTATCAA*
SAK_1911	-	PTS system mannose/fructose/sorbose family IIA subunit	2,91	5,07E-04	Pos=-108	TTTAAACCGCTTTCAT
					Pos=-65	TAGTAAAGGTTATCTT*
SAK_1919	-	Maltose 6'-phosphate phosphatase	4,31	4,09E-19		
SAK_1920	*ptsG*	PTS system, glucose-specific IIABC component, putative	4,36	1,66E-10	Pos=-92	AAGTAAGGGTTTACAA
SAK_2018	-	Cof-like hydrolase family protein	1,96	9,62E-06	Pos=-20	AAGTAAGCGCGTACAA*
**Fatty acids, cofactors and lipids metabolism**						
SAK_0419	*acpP*	acyl carrier protein	-1,51	3,64E-02	Pos=-140	AACAAACCGTTCTCAT*
SAK_0863	*lgt*	prolipoprotein diacylglyceryl transferase	2,14	2,74E-05		
SAK_0864	-	General stress protein	1,28	2,13E-02		

SAK_0877	-	HAD-superfamily hydrolase, subfamily IA, variant 1 family protein	-1,35	2,45E-02		
SAK_1166	-	ApbE family protein	2,03	4,66E-02	Pos=-36	TAGAAAACGGTATCAT
SAK_1167	-	NADPH-dependent FMN reductase domain-containing protein	2,47	1,44E-03		
SAK_1168	-	NADPH-dependent FMN reductase domain-containing protein	3,71	4,81E-08		
SAK_1169	-	formate/nitrite transporter family protein	1,81	2,68E-03	Pos=+42	TATAAAGCGCTTCCTT*
SAK_1435	-	PAP2 family protein	1,53	4,52E-02	Pos=+17	TAGTAAACTTATTCAA*
SAK_1436	*ribU*	Riboflavin transporter RibU	1,64	3,64E-02	Pos=-390	TTGAAAAAGTTTATAA*
SAK_1736	-	FMN-binding protein	3,69	3,88E-12	Pos=+160	TTGAGATCATTTTCAA*
SAK_1826	*gyaR*	D-isomer specific 2-hydroxyacid dehydrogenase family protein	1,14	3,79E-02		
SAK_1827	-	hypothetical protein	2,87	3,16E-06	Pos=-171	ATGTAAACGCTTGCTT*
SAK_1853	*ahpC*	alkyl hydroperoxide reductase, subunit C	2,68	1,72E-06	Pos=-77	ATGTAAACGTTTTTAT
SAK_1854	*ahpF*	NADH dehydrogenase	2,75	2,87E-05		
**Nucleic acids metabolism**						
SAK_0481	*rnpA*	ribonuclease P	-1,28	4,21E-02		
SAK_0950	*udk*	uridine kinase	-1,62	1,44E-02	Pos=-88	TTTAGAGCGTTTTCAA
					Pos=-219	AAATAAACTTTTTCAA*
SAK_1172	*guaC*	guanosine 5'-monophosphate oxidoreductase	-1,53	5,44E-03		
SAK_1180	*relP*	(p)ppGpp synthase	-1,24	1,50E-02		
SAK_0932	*prsA*	foldase protein PrsA	-2,13	3.37E-03		
SAK_1901	*cpdB*	bifunctional 2',3'-cyclic nucleotide 2'-phosphodiesterase/3'-nucleotidase precursor protein	1,97	1,12E-04	Pos=-77	GATTAAACGTTTTCAA
SAK_1979	-	MagZ family protein	-1,70	1,15E-02		

**Regulators**						
Two component system						
SAK_0249	*lytR/lytT*	DNA-binding response regulator	1,49	1,54E-02		
SAK_1080	*ciaR*	DNA-binding response regulator CiaR	-1,27	1,99E-02		
SAK_2066	-	sensor histidine kinase, putative	5,59	2,16E-08	Pos=-52	TAGATAGCGCTTTCAA
SAK_2067	-	response regulator	5,25	1,93E-06	Pos=+58	TTGAAATCTTCTTCAA*
Transcriptional regulators						
SAK_0075	-	RpiR family phosphosugar-binding transcriptional regulator	4,04	4,11E-13	Pos=-38	TGTTAAGCGCTTTCTT
SAK_0331	-	ECF subfamily RNA polymerase sigma factor	-2,51	3,98E-02		
SAK_0833	*ccpA*	catabolite control protein A	-3,16	7,22E-10	Pos=-74	TTGAAAGTGTTTCCAA
SAK_1034	-	GntR family transcriptional regulator	-1,59	6,55E-03		
SAK_1667	-	MerR family transcriptional regulator	-2,03	1,60E-02		
SAK_2012	-	GntR family transcriptional regulator	1,90	1,13E-02	Pos=-73	AAGAAAACGTTTTATT**
					Pos=-92	TTGTAAACGCTTTTAT
**Mobile elements**						
Prophage LambdaSa03						
SAK_0616	-	hypothetical protein	-2,04	2,40E-02		
SAK_0617	-	prophage LambdaSa03, helicase, putative	-2,21	7,40E-03		
SAK_0619	-	bacteriophage resistance protein	-2,17	1,67E-02	Pos=+187	TTGAAGTCGTTTAAAA*
SAK_0620	-	hypothetical protein	-2,63	2,83E-02		
SAK_0621	-	hypothetical protein	-2,72	4,10E-02		
SAK_0624	-	hypothetical protein	-3,82	1,65E-02		
SAK_0625	-	hypothetical protein	-2,62	1,81E-02		

SAK_0626	-	hypothetical protein	-2,64	4,16E-02		
SAK_0631	-	hypothetical protein	-2,74	3,37E-03		
SAK_0635	-	prophage LambdaSa03, terminase, large subunit, putative	-1,60	1,96E-02		
SAK_0636	-	hypothetical protein	-3,00	5,18E-03		
SAK_0637	-	prophage LambdaSa03, structural protein, putative	-2,85	4,01E-04		
SAK_0638	-	hypothetical protein	-2,36	2,01E-02		
SAK_0639	-	hypothetical protein	-2,35	1,44E-02		
SAK_0640	-	hypothetical protein	-2,78	7,32E-03		
SAK_0644	-	hypothetical protein	-2,52	2,01E-02		
SAK_0645	-	prophage LambdaSa03, pblA protein, internal deletion	-2,04	1,02E-02	Pos=+250	TTGAAAAGGGTTTCTA*
SAK_0646	-	prophage LambdaSa03, tail component, putative	-2,08	8,62E-03		
SAK_0647	-	prophage LambdaSa03, minor structural protein, putative	-2,00	1,37E-02		
SAK_0648	-	prophage LambdaSa03, minor structural protein, putative	-1,83	6,14E-03		
SAK_0649	-	hypothetical protein	-3,65	3,37E-03		
SAK_0652	-	prophage LambdaSa03, holin	-3,40	3,15E-02		
prophage LambdaSa04						
SAK_0723	-	hypothetical protein	2,78	2,45E-07	Pos=-44	TTGTAAACGCTTTTAA*
SAK_0724	-	hypothetical protein	5,84	5,68E-17	Pos=+10	TTGAAAAAGTTGACAT*
SAK_0725	-	prophage LambdaSa04, DNA polymerase	5,03	6,88E-14	Pos=-195	TTGAAACAATTTTCTT*
					Pos=+94	TTGAAATCCTTCTCTT*
SAK_0726	-	hypothetical protein	5,65	1,80E-11		
SAK_0727	-	hypothetical protein	5,21	6,90E-11		
SAK_0728	-	hypothetical protein	5,63	8,72E-13		

SAK_0729	-	hypothetical protein	5,22	1,30E-15		
SAK_0730	-	prophage LambdaSa04, DNA primase	4,94	1,27E-20		
SAK_0731	-	hypothetical protein	4,70	5,18E-03		
SAK_0732	-	hypothetical protein	4,01	3,39E-05		
SAK_0733	-	prophage LambdaSa04, SNF2 family helicase	5,50	8,46E-20		
SAK_0734	-	hypothetical protein	4,94	6,26E-13		
SAK_0736	*metK*	S-adenosylmethionine synthetase	5,14	6,42E-13		
SAK_0737	-	prophage LambdaSa04, HNH endonuclease family protein	4,78	2,29E-12		
SAK_0738	-	prophage LambdaSa04, DNA methylase	4,84	6,94E-13		
SAK_0739	-	prophage LambdaSa04, methyltransferase C-5	4,93	6,88E-14		
SAK_0740	-	hypothetical protein	3,50	3,52E-05		
SAK_0741	-	prophage LambdaSa04, P27 family terminase small subunit	3,31	5,46E-04		
SAK_0742	-	prophage LambdaSa04, terminase, large subunit	5,30	2,52E-12		
SAK_0746	-	prophage LambdaSa04, HK97 family portal protein	4,24	5,98E-16		
SAK_0747	-	prophage LambdaSa04, ClpP endopeptidase	4,35	1,30E-10		
SAK_0748	-	prophage LambdaSa04, HK97 family major capsid protein	4,80	5,83E-13		
SAK_0749	-	hypothetical protein	5,73	2,90E-09		
SAK_0750	-	prophage LambdaSa04, head-tail adaptor, putative	5,52	1,17E-11		
SAK_0751	-	hypothetical protein	4,37	7,32E-12		
SAK_0752	-	hypothetical protein	4,82	4,75E-07		
SAK_0753	-	prophage LambdaSa04, phi13 family major tail protein	4,75	7,00E-12		
SAK_0754	-	hypothetical protein	4,62	2,14E-07		
SAK_0755	-	hypothetical protein	4,38	8,13E-08		

SAK_0756	-	prophage LambdaSa04, TP901 family tail tape measure protein	5,20	1,25E-17		
SAK_0757	-	prophage LambdaSa04, tail protein, putative	4,57	1,22E-08		
SAK_0758	-	prophage LambdaSa04, minor structural protein	4,75	9,51E-15		
SAK_0759	-	prophage LambdaSa04, minor structural protein, putative	5,20	2,53E-12		
SAK_0760	-	prophage LambdaSa04, holin	3,83	4,49E-05		
SAK_0761	-	prophage LambdaSa04, mannosyl-glycoprotein endo-beta-N-acetylglucosamidase family protein	3,70	6,23E-05		
SAK_0762	-	prophage LambdaSa04 LysM domain-containing protein	4,61	1,73E-07		
SAK_0764	-	prophage LambdaSa04, site-specific recombinase resolvase family protein	1,85	4,10E-02		
IS1381						
SAK_0523	-	PTS system IIA domain-containing protein	3,27	2,32E-09	Pos=-75	TAGAAAACGTTTTCTT*
SAK_0528	-	PTS system, galactitol-specific IIA component, putative	3,37	4,75E-04	Pos=-44	ATGGAATCGATTTCAT*
SAK_0529	-	PTS system, galactitol-specific IIC component	2,76	3,31E-03		
SAK_0531	-	AraC family transcriptional regulator	2,19	3,59E-04	Pos=-28	AAGTAAACGCTTTAAT*
					Pos=-147	ATGTAAGCGCATACAT*
SAK_0532	-	sugar ABC transporter, sugar-binding protein, putative	3,93	2,60E-05	Pos=-196	AAGTAAACGCTTTAAT*
					Pos=-411	AAGAAACCGCTCACAA*
					Pos=-77	ATGTAAGCGCATACAT*
SAK_0533	-	sugar ABC transporter, permease protein, putative	4,15	3,30E-05		
SAK_0534	-	ABC transporter, permease protein	3,33	1,02E-04		
SAK_0535	-	alpha-galactosidase, putative	4,09	4,13E-10		
SAK_0537	*galT*	galactose-1-phosphate uridylyltransferase	3,84	5,45E-06		

SAK_0538	*galE*	UDP-glucose 4-epimerase	2,53	3,65E-03		
SAK_0539	-	aldose 1-epimerase, interruption-N	2,41	9,19E-03		
**Virulence**						
SAK_1891	-	sialidase	-2,17	6,35E-03		
SAK_1892	-	sialidase N-Ter	-3,77	7,66E-04		
SAK_1991	*cspA*	cell surface serine endopeptidase CspA	-1,75	1,22E-02		
**Stress**						
SAK_0089	-	MATE efflux family protein	2,24	7,67E-06		
SAK_0250	*lrgA*	LrgA/CidA family protein	1,32	2,01E-02	Pos=-72	AATAAAACGTTTTCAT
SAK_0251	*lrgB*	LrgB/CidB family protein	2,24	1,08E-03		
SAK_0348	-	pyridine nucleotide-disulfide oxidoreductase	4,03	1,48E-14	Pos=-46	TTGTTACCGTTTTCAA
SAK_0349	-	hypothetical protein	3,90	5,75E-15		
SAK_0902	*cshA*	DEAD-box ATP dependent RNA helicase	-1,57	2,81E-02	Pos=-142	ATGAAAGCGTGTTATT
SAK_1110	*cstA*	carbon starvation protein CstA	3,79	6,46E-09	Pos=-50	ATGAAAACGCTTAATA
SAK_1217	*tpx*	thiol peroxidase	1,80	1,57E-02		
SAK_1233	*cidA*	LrgA/CidA family protein	-2,75	3,18E-04	Pos=-66	ATGTAAACGTTTCATA
SAK_1234	*cidB*	LrgB/CidB family protein	-2,73	8,95E-06		
SAK_1352	-	Glutathione S-transferase domain protein	1,93	4,14E-03	Pos=-9	TTGTCATCGTATACTT*
SAK_1689	*uspA*	hypothetical protein	4,91	1,50E-08	Pos=-144	ATGAAAGCGTTTTATG*
**Unknown functions**						
SAK_0012	-	hypothetical protein	-2,20	1,29E-04		

SAK_0273	-	hypothetical protein	-1,14	4,61E-02		
SAK_0432	-	hypothetical protein	1,14	4,11E-02		
SAK_0442	-	hypothetical protein	-1,30	2,88E-02		
SAK_0507	-	hypothetical protein	-3,28	2,25E-02	Pos=-327	AAGAAATTGCTGACAT
SAK_0546	-	hypothetical protein	-1,36	1,81E-02		
SAK_0705	-	hypothetical protein	-2,14	7,69E-03		
SAK_0769	-	hypothetical protein	2,82	8,58E-08	Pos=+194	TTGAAGTCCCTTTCTT
SAK_0802	-	hypothetical protein	-3,82	2,12E-03	Pos=-90	TTAAAATCGTTTTCAG
SAK_0805	-	hypothetical protein	-2,50	1,53E-02		
SAK_0923	-	hypothetical protein	1,78	2,87E-02		
SAK_0957	-	hypothetical protein	2,32	3,60E-03	Pos=-47	ATGACAAGGCTTACAT
SAK_1109	-	hypothetical protein	-1,33	1,41E-02		
SAK_1130	-	hypothetical protein	-2,59	1,86E-02		
SAK_1131	-	hypothetical protein	-3,04	4,75E-02		
SAK_1340	-	hypothetical protein	1,74	2,03E-02		
SAK_1367	-	hypothetical protein	1,37	3,93E-02		
SAK_1530	-	hypothetical protein	1,30	1,91E-02		
SAK_1640	-	hypothetical protein	-1,41	4,33E-02	Pos=-95	ATGTAGTCGTTAACAA*
SAK_1806	-	hypothetical protein	-2,45	1,37E-02		
SAK_1807	-	hypothetical protein	-2,35	4,22E-03	Pos=-211	ATAAAGACGCTTTCAA*
SAK_1842	-	hypothetical protein	-2,83	1,26E-02		
SAK_1913	-	hypothetical protein	1,30	3,53E-02		
SAK_1940	-	hypothetical protein	-1,58	1,12E-02		

SAK_1952	-	hypothetical protein	3,42	3,64E-03		
SAK_1953	-	hypothetical protein	2,44	1,57E-02	Pos=-490	AAGAAAGCGTTATCTT*
SAK_2063	-	hypothetical protein	3,41	2,37E-08		
SAK_2077	-	hypothetical protein	-1,70	2,27E-02		
SAK_2113	-	hypothetical protein	-1,74	0,028243		

Pathway predictions and gene annotations were conducted with the Kyoto Encyclopedia of Genes and Genomes (KEGG Pathway) (https://www.kegg.jp/kegg/) (5) and the MicroScope platform (https://www.genoscope.cns.fr/agc/microscope) (6), as explained in the Materials and methods section. Experiments were performed over three independent biological replicates.

REFERENCES

1. Tettelin H, Masignani V, Cieslewicz MJ, Donati C, Medini D, Ward NL, Angiuoli SV, Crabtree J, Jones AL, Durkin AS, DeBoy RT, Davidsen TM, Mora M, Scarselli M, Ros IM y, Peterson JD, Hauser CR, Sundaram JP, Nelson WC, Madupu R, Brinkac LM, Dodson RJ, Rosovitz MJ, Sullivan SA, Daugherty SC, Haft DH, Selengut J, Gwinn ML, Zhou L, Zafar N, Khouri H, Radune D, Dimitrov G, Watkins K, O'Connor KJB, Smith S, Utterback TR, White O, Rubens CE, Grandi G, Madoff LC, Kasper DL, Telford JL, Wessels MR, Rappuoli R, Fraser CM. 2005. Genome analysis of multiple pathogenic isolates of *Streptococcus agalactiae*: Implications for the microbial "pan-genome." PNAS 102:13950–13955.

2. Moulin P, Rong V, Ribeiro E Silva A, Pederick VG, Camiade E, Mereghetti L, McDevitt CA, Hiron A. 2019. Defining the role of the *Streptococcus agalactiae* Sht-family proteins in zinc acquisition and complement evasion. J Bacteriol 201:e00757-18.

3. Biswas I, Gruss A, Ehrlich SD, Maguin E. 1993. High-efficiency gene inactivation and replacement system for gram-positive bacteria. J Bacteriol 175:3628–3635.

4. Poyart C, Trieu-Cuot P. 1997. A broad-host-range mobilizable shuttle vector for the construction of transcriptional fusions to beta-galactosidase in gram-positive bacteria. FEMS Microbiol Lett 156:193–198.

5. Kanehisa M, Sato Y, Kawashima M. 2022. KEGG mapping tools for uncovering hidden features in biological data. Protein Science 31:47–53.

6. Vallenet D, Calteau A, Dubois M, Amours P, Bazin A, Beuvin M, Burlot L, Bussell X, Fouteau S, Gautreau G, Lajus A, Langlois J, Planel R, Roche D, Rollin J, Rouy Z, Sabatet V, Médigue C. 2020. MicroScope: an integrated platform for the annotation and

exploration of microbial gene functions through genomic, pangenomic and metabolic comparative analysis. Nucleic Acids Research 48:D579–D589.
